# Spirit of the English Periodicals, and Notices of English Medical Literature

**Published:** 1835-10-01

**Authors:** 


### Periscope;

OR,

### CIRCUMSPECTIVE REVIEW.

"Ore trahit quodcunque potest, atque addit acervo."

I.

### Spirit of the English Periodicals, and Notices of English Medical Literature.

### Cases of Carditis, with Remarks. By Dr. Stroud.*

We notice these cases, because their author appears to indulge in more than one assumption, which require comment.

*Case.* W. D. æt. 53, applied at the Northern Dispensary in May, 1831, on account of dry cough, disturbed sleep, pain at the epigastrium and in the arms, and headache. He had been a sailor, a blacksmith, a shoemaker, and a drunkard. The symptoms had existed for three years. We need not detail the treatment. The prescriptions are given at great length, and though very good in their way, we do not deem it necessary to transcribe them. On examination with the stethoscope, "the respiratory murmur was tolerably audible, but the sound of the heart's action was dull, while the pulse at the wrist was strong." To all acquainted with auscultation we need hardly say that this account of the auscultic phenomena is not satisfactory.

The hands and feet became œdematous, the cough more troublesome and attended with expectoration; orthopnœa was established, and, in spite of the most various prescriptions, the patient sank, and on the 31st of May he died. We give the notes of the appearances discovered after death, without abbreviation.

"*Post-mortem Appearances.* An inspection having been obtained the next day, the following appearances were observed.

*General Conditions.* The body was robust and well formed, exhibiting little œdema, and no fat. The skin was firm, and the muscles were thick and red. The head and spine were not examined.

*Thorax.* The cartilages of the ribs were nearly ossified, especially those of the sixth pair. The mediastinum was broad, and its cellular membrane dense. The right pleural sac contained a considerable quantity of reddish serum; and owing, perhaps, to the weight of this liquid, and of the diseased liver, descended deeper into the abdomen than its fellow, contrary to the usual occurrence. The inner surfaces of the left pleural sac were universally united by short and firm adhesions, apparently of old standing. The right lung was somewhat inflated, or emphysematous, but otherwise healthy. The left lung was much hepatized, more especially the central parts, which were dense and red, while those near the surface were tolerably sound and spongy. The distinction was very perceptible on pressing the several portions between the fingers, when the one was found crepitous, and the other fleshy. There was no tubercle, vomica, or extravasation, either in the parenchyma of the lungs, or in the bronchial tubes: but the bronchial membrane, which was of a deep red colour, was coated with a little frothy cream-like mucus, and the mesochondriac fibres were very distinct.

The pericardium, with its contents, was of nearly double the usual size. The membrane itself was rather thick-

* Med. Quarterly Review, No. VIII.

ened, and contained several ounces of pellucid watery liquid. The substance of the heart was, in general, firm, thick, and red, evidently from the influence of chronic inflammation. The cavities on both sides contained a good deal of soft, red, and broken coagulum. The right auricle was much dilated. Its musculi pectinati were extensive, but loose, exhibiting the appearance of strong fleshy columns, with wide interstices. The right ventricle presented nothing remarkable. The left auricle was dilated, and somewhat thickened. Its auricula propria was singularly contracted, and convoluted in a spiral form, like that of a cornu Ammonis, but quite pervious to the apex. The walls of the left ventricle were very thick. The aorta was much diseased, and, to some distance from the heart, was of nearly double its ordinary size and substance. On slitting it up, the lining membrane was found hypertrophied, presenting a scabrous and irregular surface, roughened by decussating fibres, with intervening indentations and depressions. The aortic valves were somewhat thick and rigid, but less so than the opposite lining membrane, which was slightly ossified. All the other valves, both cardiac and arterial, were sound. The orifices of the coronary arteries were contracted, and not easily discoverable, being concealed by a thin transverse fold, resembling an irregular valve.

*Abdomen.* The peritoneal sac contained a moderate quantity of reddish serum. The omentum was voluminous, and extensively spread over the surface of the intestines, but free from disease, and almost destitute of fat. The stomach was inflated, and of large size. Its lining membrane was of a dark colour. The intestines were generally healthy, but some portions of the ileum were red and contracted. The colon at the sigmoid flexure was abruptly and considerably dilated, but, with this exception, the large intestine was throughout much contracted and indented, containing only a small portion of thin yellow fæces. The liver was, apparently, of nearly double its usual size and weight. On making incisions into its substance, it was found to be universally dense, and uniformly mottled with red and white spots, like some varieties of porphyry. The gall-bladder was small, and inclosed a little dark-coloured and ink-like bile. The spleen, and the kidneys were firm and sound; as were likewise the mesentery, and the urinary bladder, which was empty and collapsed."

We have given this account of the examination after death entire, because it allows us to make some observations on the subject. The narrator of a case should select the important particulars for the public. He should *study* symptoms generally and minutely, in order to discover what are and what are not material. But the same minuteness should never be carried into a report, where the object is to set before the reader the results of the writer's observation and inquiries, not every item of the inquiry itself. Dr. Stroud, however, thinks the public will be anxious to learn that the skin was firm, and the muscles red; and, the patient being 53 years of age, he is particular in specifying that the cartilages of the ribs, especially of the sixth, were nearly ossified—he observes that the mediastinum was broad—that there was coagulum in the heart—that the "auricula propria" of the left auricle was convoluted like a "cornu ammonis"—that the omentum was voluminous and spread over the intestines—that the stomach was large—that the spleen, kidneys, mesentery, and urinary bladder were healthy, with a variety of other circumstances equally irrelevant and unimportant. The fact is, that Dr. Stroud could have said all that was necessary, with reference to the dissection, in one quarter of a page, though nearly a page and a half are occupied with the details we have in part alluded to.

We said that Dr. Stroud indulged in some assumptions. He observes that "the substance of the heart was in general thick, firm, and red, evidently from the influence of chronic inflammation." The propriety of the "*evidently*" is by no means so evident to us. On the contrary, the evidence is in favour of the existence of hypertrophy of the heart, independently of any positive carditis. Sure we are that if increased firmness, thickness, and redness are to be deemed sufficient to prove that inflammation of the substance of the heart was going on, that disease would be more frequent than the best pathologists suspect it to be. The great number of cases of ventricular hypertrophy display the characters in question, characters, be it observed, displayed by all muscles in the hypertrophic condition.

We subjoin a specimen of our author's mode of reasoning.

"This conjunction of hypertrophy with dilatation, which existed not only in the heart, but, also, in the aorta to some distance from it, may serve to show, in opposition to the doctrine of certain eminent authors, that inflammation depends not on mere debility in the part affected, but on a specific irritation, accompanied with various kinds and degrees of functional derangement. The extensive development of the musculi pectinati, and the remarkable convolution of the left auricula propria, owing, apparently, to a permanent contraction of its spiral muscular fibres, sufficiently prove that the expansion of their cavities was not the result of a simple deficiency of power. The increased and irregular growth of the lining membrane of the aorta had somewhat affected its valves, producing a tendency to ossification, and a partial obstruction of the orifices of the coronary arteries, which, probably, contributed to diminish the violence, and to retard the progress of the disease. This morbid state of the aorta is a frequent consequence of habitual intemperance, especially, perhaps, of indulgence in ardent spirits, and is often the primary cause of cardiac disease, which in this case seems, however, to have been chiefly independent."

The circumstance that such a disease as hypertrophy and dilatation of the heart occurs, is not of itself, we apprehend, sufficient to overthrow the doctrine of debility in the part in a state of inflammation. Dr. Stroud, however, is more sanguine, and we will not venture to molest him in his anticipations. We are by no means confident that enlargement of the aorta tends to occasion disease of the heart; but we are quite sure that the converse is correct, and that hypertrophy of the left ventricle very frequently gives rise to dilatation of the aorta. One more quotation and we have done.

"The dropsical state usually attendant on such complaints, had not proceeded to any extent, either in the abdomen, or in the common cellular texture, having been chiefly confined to the serous membranes near the seat of the disease, namely, the pericardium, and the right pleural sac. Its limited amount might be ascribed to the early failure of vital power, and to the original soundness of the constitution, to which, also, in conjunction with the hypertrophy of the heart, it was probably owing that the solid viscera of the abdomen were generally firm, and the liver, in particular, was much enlarged and condensed, exhibiting on section a mottled texture, like that of porphyry, the joint result, perhaps, of intemperance, and of cardiac disease. The increased development of the stomach, the dark colour of its lining membrane, and the redness and constriction of some of the intestines, were, no doubt, partly dependent on the obstruction of their circulation, occasioned by the morbid state of the liver and heart."

We doubt if any comment is required to point out the improbable theories contained in this passage. How the limited extent of the dropsy could reasonably be imputed to the early failure of vital power, we profess ourselves unable to explain. Neither do we perceive why hypertrophy of the heart should tend to make the solid viscera of the abdomen firm. And, finally, why the obstructed state of their circulation should render the stomach large and dark, and the bowels red and small, is beyond our comprehension.

The second case is possessed of more intrinsic interest than the preceding, or than that which follows it.

*Case.* In April, 1833, a young woman, about 22 years old, was delivered of a still-born male child, at the full period. She seemed much exhausted, was in a wretched state of want, complained much of thirst, had a pale countenance, a small and very quick pulse, and in thirty-six hours after delivery she died. It appeared upon inquiry that she had led an irregular and intemperate life, and that she had been abroad during the whole day preceding that on which her labour had occurred. From a very early age she had been subject to pains in the region of the heart, attended with palpitation and a sense of weight in the chest, and likewise to rheumatic pains in the shoulders and the arms.

*Dissection.* Passing over unimportant particulars, we may mention the following as the principal appearances observed :—

The right pleural sac contained more than a pint of red serum; the left was nearly filled up by adhesions. The lungs were tolerably sound. The heart was so dilated and hypertrophied as to be nearly three times its natural size.

" The two layers of the pericardium, universally and firmly adherent, were fleshy, rough, and of a deep red colour; and, at one spot on the upper part of the right ventricle more than an inch in diameter, was a thick cartilaginous deposit verging to ossification. The inferior vena cava was very large and turgid, but all the other vascular trunks seemed to have been compressed and reduced in size, by the contractile force of the inflamed pericardium; whilst, in consequence of the enlargement of the entire organ, their position was, at the same time, somewhat altered. All the cavities of the heart were to a considerable extent thickened and dilated, and they all contained much blood, chiefly in the state of soft, loose, dark red coagulum, intermixed with some portions which were paler, and of a firmer consistence. The lining membrane of the auricles, especially that of the left, was white and opaque, as if from an interstitial deposit of albumen. The left ventricle, which was the principal seat of hypertrophy, preserved its expanded form and dimensions when cut open, and its fleshy columns were very prominent and distinct. The cardiac valves, on both sides, but particularly the mitral, were somewhat thickened and opaque, from incipient interstitial deposit, tending in some points to ossification. The aortic valves were in a similar state, but not to the extent of interrupting their function."

We need not add Dr. Stroud's remarks. One is so obvious as to strike every one who reads the case—the compatibility of so much enlargement of the heart with the pursuit of dissolute and intemperate pleasures. Yet this is no more than we frequently observe. Persons with extreme organic alteration of the heart, go through the avocations, and enjoy in some degree the amusements of the world. Attila, and many others, down to the late Irish comedian Connor, have died in the act of venereal intercourse, in consequence of the rupture of aneurism, or of great disease of the heart. The late Sir William Douglas walked in from Kensington to see us on the morning of the day on which he died, and there was not a symptom then observable to lead the most experienced physician to anticipate so speedy a demise.

There is one feature in the case before us which should not be altogether overlooked. The two layers of the pericardium were universally adherent. Now this patient had suffered from rheumatic symptoms, and perhaps there is no cardiac alteration so frequently, we might say so constantly, coexistent and connected with rheumatism, as inflammation of the pericardium terminating in adhesion of its layers, and finally producing, or tending to produce, enlargement of the substance of the heart itself. If a patient has been subject to rheumatic affections, especially if he has laboured under acute rheumatism, and symptoms of cardiac disease are present, ten to one that disease is adherent pericardium. Such is the result of our observation.

Dr. Stroud is an active and intelligent practitioner; and these slight strictures are not intended to check his industry, but to direct his talents into the most useful channels.

---

### Fatal Dissection Wound.

Dr. Charles Benson has published an interesting but melancholy case of this kind in the last number of our Dublin contemporary. The subject was a diligent young student, who was found by Dr. B. labouring under low fever, such as students are liable to in the first season of their dissections. He was bled, and took some saline and mercurial medicines; but the progress was unsatisfactory, and on the third or fourth day Dr. B. found him in a very dangerous state, the pulse being 120—tongue brown—some delirium. On questioning his brother, it was found that he had scratched one of his fingers in dissection, and although there were no red lines running up the arm from the wound, yet there was great tenderness in the same side of the chest when pressed, as also below the axilla, about the clavicle, and the pectoral muscle. Dr. Colles was called into consultation, and it was now clear that the case was one of dissection-wound. The symptoms, in spite of judicious treatment made head—matter formed below the axilla. It was discharged by a puncture, but without relief. The restlessness and delirium increased, and the unfortunate youth died comatose, seven days from the time he began to complain. The affected side was swelled, and under the pectoral muscle was found some healthy pus, not contained in a cyst, but diffused through the cellular membrane. The lungs were healthy on both sides.

"In the right pleura a small quantity of bloody serum existed. The heart was healthy, but the pericardium contained more serum than usual. In the head the dura mater was sound; the cerebral layer of the arachnoid was opaque over the anterior lobes, but exhibited no mark of recent inflammation; the substance of the cerebrum and cerebellum was every where healthy, in the ventricles, however, half an ounce of colourless serum was found, but no other sign of disease."

Dr. B. brought this case before the Surgical Society of Dublin, because the formidable disease of which it is a specimen, appears to be still imperfectly understood. Dr. B. thinks there are not cases enough on record; but that the communications of individuals will ultimately lead to the formation of a *corpus historicum* from which inferences may be fairly drawn. To Dr. Colles, Dr. Duncan, and Mr. Travers, the profession is under obligations. Our readers are aware of the general impression that the danger from dissection wounds is in proportion to the freshness of the subject. Some writers have averred that the peculiar constitutional fever of a poisoned wound has never resulted from the dissection of *putrid* bodies. In the above case, however, the subject was very putrid. The following are the grades and varieties of poisoned wounds from dissection, which our author has observed.

"1st. A small pustule, unattended with much pain, confined to the skin, and disappearing in a few days.

2nd. A chronic inflammation, confined to a point under the skin, causing little or no uneasiness, not suppurating, but leaving a very small hard tumor, which after remaining many months, gradually disappeared.

3rd. An erysipelatous inflammation around the wound, which slowly creeps along the finger to the hand, and remains for two or three weeks wandering about the fingers, after the wound has been quite healed.

4th. Violent inflammation of the injured part, with intense pain, followed by sloughing of the skin and cellular tissue in the immediate vicinity.

5th. Inflammation of the sheaths of the tendons, as in severe paronychia.

6th. Inflammation not confined to the part, but running along the superficial absorbents. The glands in the axilla suppurate. There is much local and constitutional suffering.

7th. The deep absorbents appear to be engaged. Some red lines may be observed on the hand; we lose them in the arm, and again we find them in the axilla, with inflamed glands going on to suppuration; high fever; intense pain.

8th. Lastly, as in Mr. J.'s case, the constitutional symptoms show themselves before the local. Fever of the typhoid character. No sign of active inflammation in the wound, but a vesicle or pustule often forms on it. Absorbents not inflamed, but there is diffused inflammation and suppuration in the cellular tissue of the pectoral and axillary regions.

This last case heretofore appeared to me to depend on the absorption of a *peculiar* animal poison, generated, in some way, at or about the time of death, and losing its specific virulence when putrefaction occurred. The others, I conceived, arose from an irritating substance of no specific nature, and assumed their different characters from the texture wounded, the irritating qualities of the matter introduced, or still more from the state of the patient's health at the time of the injury, and the peculiarities of his constitution."

Our author asks what is the plan of treatment which should be adopted in cases like the one here related? Should it be the stimulant or the antiphlogistic?

---

### Dr. Graves' Remarks on Dropsy, with a Case in Illustration.

"One of the patients, J. Austin, states he has been ill two months before he came into hospital, and acknowledges that his illness was the result of long continued habits of inebriety. Careless and intemperate in his mode of life, and frequently exposed to cold and wet, he got an attack of bronchitis, accompanied by a sense of constriction about the chest and difficulty of breathing. He was bled for this, and states that the bleeding relieved his dyspnœa; but about this period he remarked that an anasarcous swelling appeared in his face, neck, and chest.

In this case, gentlemen, we have a specimen of the ordinary history of dropsy in this country:—first, intemperate habits, next exposure to cold, followed by bronchitis or pneumonia, and then dropsy commencing in the face, chest, and upper extremities. I have on a former occasion pointed out to the class the importance of observing in what part of the body dropsical swelling first appears, because, by doing so, we obtain a more accurate idea of its nature, and are furnished with a clue towards discovering its source. Dropsy is generally the consequence of organic disease of some deep-seated viscus. When it is produced by thoracic disease, as bronchitis, pneumonia, or affections of the heart, it is said that the swelling always begins in the face, neck, trunk, and upper extremities; when it depends upon chronic hepatitis, disease of the spleen, obstruction of the system of the vena porta, or disease of the mesenteric glands, the swelling commences in the abdomen, and then proceeds to the lower extremities; but when it arises from mere debility, the consequence of hectic fever, long-continued diarrhœa, or a cachectic state of the system, the effusion is first observed in the lower extremities, coming on in the evening, and again disappearing towards morning. The history of dropsical swellings, therefore, by informing us in what part they first appeared, is often sufficient to indicate the general nature of the producing cause.

When this man came into the hospital, his cough had disappeared, and there was not any unequivocal symptoms of disease of the heart, but he had considerable dropsical swelling of the face, chest, and superficial parts of the abdomen; his appetite was bad, and on examining his urine we found it loaded with albumen, and of the specific gravity of 1029. Though he had no fever or dyspnœa at the time, we commenced the treatment by general bleeding, because he was a person of rather robust constitution, and on account of his dropsy having originated in cold. In persons who are able to bear bleeding, and where the disease has commenced in an acute form, you may often commence the treatment of dropsy by a single bleeding with great advantage, even though there be no fever or local inflammation present. We next prescribed an aperient injection, to be followed by a vapour bath.

I then, by way of trial, gave him an electuary containing some diaphoretic medicines, and found that it acted well on the skin, and that sweating could be easily induced. This furnished me with a key to the after treatment. Whenever you find that sweating can be easily brought on in dropsical cases, you should obey the hint given by nature. You should not under such circumstances have recourse to mercury, or hydragogue purgatives, or diuretics; you are to open the passage which nature has pointed out, you are to encourage diaphoresis, and you may rely upon it that you will in this way effect an easier, safer, and more permanent cure than you could by any of the various modes employed for similar purposes. We therefore gave this man a powder containing four grains of Dover's powder and five of nitrate of potash three times a day. The Dover's powder is tempered by combining it with nitrate of potash, which is an antiphlogistic, and prevents the former from exercising a heating effect on the system. Having continued these powders for seven or eight days, we commenced the exhibition of opium in doses of half a grain four times a day, to be increased after a few days to half a grain every fourth hour. Under the use of vapour baths used daily, and opium to the amount of three grains in the twenty-four hours, the man has improved wonderfully, and the dropsical swelling is fast subsiding. Opium has here, you may have remarked, produced no bad effects. The tongue is neither dry nor furred, and it has not any of that appearance which is observed in persons who are in the habit of taking opium; his appetite is unimpaired, his bowels regular, and his strength undiminished.

Now why did I give opium in this case? The more advanced students will perceive that I have treated it nearly in the same way as I treat cases of diabetes; because I have taught, and have been the first to teach, there seems to be an analogy between chronic dropsy and diabetes, and because experience has proved to me that this mode of treatment is most likely to be attended with success. I shall not dwell on this point at present, as I have already published a paper on it in the *Dublin Medical and Chemical Journal*, to which I refer you, merely observing here, that opium and other diaphoretics increase strength, remove the dropsical swelling, diminish the quantity of albumen in the urine, and bring on convalescence without producing any bad effects on the head or digestive system. Dr. Osborne, Dr. Gregory, and Dr. Bright have asserted that the presence of albumen in the urine arises from a particular disease of the kidney, in which the whole texture of the organ is altered, it becomes hypertrophied, finally harder than natural, and of a pale yellowish colour. On the other hand, Dr. Elliotson, Dr. M'Intosh, and myself, have opposed this view of the question. It is true that this kind of kidney is sometimes found to exist with an albuminous state of the urine, but this is by no means invariably the case. I have seen many cases of albuminous urine which yielded completely to the exhibition of opium, and this surely could not happen if organic disease were present. And though the cases in which this has occurred are not very numerous, still the evidence is good, and it cannot be denied that such a state of the urinary discharge may and does depend on constitutional causes totally independent of disease of the kidneys. I have very little doubt that it is to such an origin the present case is to be referred, and I feel confident that we shall cure it with opium. I am anxious that you should attend to this case and watch the result, for the treatment is quite different from that employed by others. I say this without meaning to claim any originality, but I may be permitted to say that it is a mode differing very much from those generally pursued. It cannot be used in cases where fever or local inflammation is present, but when the local and general excitement has been subdued, or when the case is chronic and unaccompanied by quick pulse, or any symptoms of visceral inflammation, it may be employed with safety and advantage."*

---

### CREOSOTE.

As this powerful stranger, which has lately made its way into therapeutics, is occasioning considerable curiosity, we shall here introduce an account of its properties, mode of preparation, and medicinal agency, from a recent edition of Dr. Gully's "FORMULARY OF NEW MEDICINES," published by Churchill.

"The name of this new remedy is derived from the Greek κρέας, flesh, and and σώζω, to preserve. It was discovered last year by M. Reichenbach de Blansko in pyroligneous acid, in the first instance, and subsequently in the different kinds of tar.

In the process which led to the discovery of creosote, M. Reichenbach found that his fingers were deprived of their epidermis, and he conjectured, from this vehement action on organic matter, that this substance might be the mummifying principle of pyroligneous acid, and might also serve an important therapeutical purpose in the living body. This explanation has since been realized.

#### *Physical Properties of Creosote.*

Creosote is an oily, colourless, transparent liquid, of a penetrating odour, resembling that of smoke, or smoked meat, and of a burning and exceedingly caustic taste. It has a specific gravity of 1,037.

#### *Chemical Properties.*

It boils at 203° Centigrade, and is not congealed by a cold of—27° C.; it burns with a strongly fuliginous flame. With water at 20°, it forms two combinations, one a solution of one part in 80, and the other of 10 parts in 100. This aqueous solution does not change turnsol, nor is it neutralized by acids or alkalis. Nitric acid causes red vapours. A small portion of concentrated sulphuric acid turns it red, but a larger quantity blackens it, the acid itself being also decomposed. Acetic acid seems to be its specific solvent, for it holds any quantity of it. All the acids, even the carbonic, separate creosote from its combinations with potass and soda, without otherwise affecting it. It dissolves a great number of salts, some with, and some without heat. Alcohol, ether, carburetted sulphur, and acetic ether combine with it in any proportion. It decomposes or dissolves resins, resinous colouring matters, and other similar substances.

Shaken with white of egg, coagulation immediately takes place. Fresh meat, soaked for an hour in a solution of creosote, and then dried, may be exposed to the sun, without fear of putrefaction; in a week it becomes hard, has the smell of smoked meat, and becomes reddish-brown. Fish may be preserved in the same manner. Birds poisoned with creosote, remain nearly two months without emitting any putrid odour.

These effects on animal matter closely resemble those of pyroligneous acid and tar-water, and demonstrate almost to a certainty that creosote is the preservative principle of those fluids. This, however, has been further shown by the extraction of creosote from both of them. I shall confine myself to the preparation of creosote from tar, as it is procured more abundantly, and by an easier process, from that substance.

#### *Preparation of Creosote.*

In the dry distillation of tar from wood, the fluid collected in the receivers contains an empyreumatic acid water, which is rejected, and oil of tar, which is placed in glass retorts and rectified. In these two distillations the oil of tar is at first light, but as the heat is increased, its gravity augments. At one period of the process the oil sinks to the bottom, and a fluid which is poor in cresote, and consists mostly of *eupione*, and other substances that interfere with the purity of the creosote, floats above it: this is poured off, and the pale yel-

---

* London Medical and Surg. Journal.

low tar-oil is heated. Carbonate of potass is added, until the carbonic acid is no longer disengaged on shaking; the mixture is decanted, in order to separate the acetate of potass, and the oil is again distilled in a glass retort, and all the first products that float on the water are rejected. The oil is then dissolved in a solution of caustic potass of the specific gravity 1,12; heat is thereby developed, and a portion of the materials composed of eupione, &c. not being dissolved, floats on the surface, and is removed. The alkaline solution is poured into an open capsule, and regularly heated to boiling. It rapidly absorbs the oxygen of the atmosphere, whereby a peculiar oxidizable principle in it is decomposed, and the mixture then turns brown. After cooling in the open air, diluted sulphuric acid is added until the oil is set at liberty. It is then distilled with water, holding a little caustic potass, and the whole is kept boiling until the quantity of oil which passes from the retort becomes diminished; at this point the distillation should cease. The oil and water in the receiver are again distilled with potass, and the same treatment with sulphuric acid repeated, as in the former instance. A third distillation is then made, and a little phosphoric instead of sulphuric acid is added, in order to take up some ammonia retained in the oil.

The oil is then for the third time dissolved in caustic potass, and if the preceding processes have been carefully managed, they combine, without leaving any residue of eupione, and the mixture, on exposure to the air, does not turn brown, but takes on a slightly reddish tint. As long, however, as any eupione remains, and the mixture turns brown, the solution in potass should be repeated. In this state, the creosote is not entirely pure, but it may be used for medicinal purposes.

It may be obtained perfectly pure by distilling it with water alone, then rectifying the product of the distillation repeatedly, until no water passes over when the heat is raised to 203° C. The last product is creosote unalloyed by eupione, picamare, water, or other matters.

M. Reichenbach endeavoured to simplify this tedious process; but he found that the product was always unfit for internal use, while its action on the surface was much impaired. So procured, its emetic effects were most violent; a single drop applied to the tongue caused in the space of a minute, excessive nausea with tremors, succeeded by vomiting, and great prostration of the powers. These effects he attributes to the presence of eupione, and he therefore recommends the process above-described to be followed on all occasions.

### *Physiological Action of Creosote.*

Applied on the tongue in a concentrated form, creosote causes violent pain, though no redness or tumefaction is present: a strong taste of smoke extends to the throat. Poured on the skin, it produces a burning sensation with rubefaction and erosion.

Flies, spiders, and small fishes die in the course of two minutes, when immersed in a solution of twelve drops of creosote, in two ounces of water.

Two drachms given in half an ounce of water to a puppy-dog induced the following symptoms; complete prostration of muscular power, drooping of the head, fixation of the eyes, vertigo, apparent stupefaction of all the senses; the respiration, from being laboured, was at the end of three minutes almost entirely stopped by an abundant secretion of viscid, filamentous mucus; to which was added vomiting of whitish milky fluid, with spasmodic contraction of the abdominal muscles. These symptoms got gradually worse for two hours, the respiration becoming more laborious, and at longer intervals, the limbs being seized with tremors, then with convulsive contractions, and the whole ending in death.

On opening the body of the animal, all the tissues of the body, except the liver, exhaled a strong odour of creosote. The mucous digestive membrane gave signs of inflammation throughout its whole extent; the matters contained in the stomach coagulated white of egg, and, heated, gave out the powerful tar-smell of creosote. In the heart and the immediate great vessels the blood appeared to be much more firmly coagulated than usual. The lungs were gorged over the greater part of their extent with reddish-brown blood; the more ruddy parts of them floated in water readily: the darker portions scarcely swam at all. No sign of congestion about the brain appeared.

On injecting equal parts of creosote and water into the carotid artery of a dog, the same symptoms were produced, but death ensued more speedily.

If concentrated or diluted creosote be added to blood, the latter thickens and becomes reddish-brown, with small spots of white, probably coagulated albumen: on further exposure to the air, the blood passes to a yellowish-red colour.

The signs of poisoning with creosote, therefore, are the redness of the gastro-intestinal mucous membrane, the peculiar thickness and colour of the blood, the property possessed by the matters in the stomach of coagulating albumen, and more especially the peculiar odour exhaled by all the tissues of the body.

Plants watered with a solution of creosote, fade and die in the course of a few days.

*Medicinal Employment.*

M. Reichenbach's first essays of his newly-discovered remedy were made on slight burns, infantile excoriations, and wounds. Subsequently he was induced to try it in extensive burns by hot iron and boiling fluids: in itch and various kinds of tetters: in gangrene consequent on extensive compound fracture of the leg: in caries of the phalanges of the fingers and toes: in tooth-ache, though it fails to put a stop to the caries of the tooth: in open, fungous whitlow; in scrofulous ulcers of the throat, leg, and joints of the fingers: in ulcerated white-swelling of the knee of two years' standing; in chancres and other syphilitic ulcers: in wounds from cutting and piercing instruments, caustic alkalis, &c., in which cases the wounds did not cure by suppuration, but by actual desiccation caused by the creosote. In all these instances he has found the remedy most effectual and astonishingly rapid in its operation. Thus in a case of old-standing and scrofulous ulceration of the throat, with purulent discharge from the ears, the ointment of creosote to the former, and the injection of creosote water into the latter, put an end to both in the course of three weeks.

Internally, M. Reichenbach has given it in several cases of hemoptysis; in two of these, the sanguineous expectoration had continued for upwards of a week, when the administration of four drops of creosote, on sugar, daily for four days, arrested the flow of blood.

Turning to the practice of the French physicians, we find that creosote has been successfully employed in burns by Berthelot and Goupil, who observe, that in treating these with creosote, the tendency to cicatrize from the circumference to the centre, and the consequent contractions and irregularities, are avoided; in various dry and moist tetters by Goupil, Coster, Berthelot, Martin-Solon, Duchesne-Duparc and Dauvergne: in chancres and old venereal ulcers, by Künckel, Lesseré, and others; in sanious ulceration of the cervix uteri, by Colombat: in a cancerous ulcer of the nose, by Breschet: in chronic inflammation with suppuration of the edges of the eyelids, by Coster: in cancer of the womb, by Hippolyte Cloquet and Tealier: in varicose, ill-conditioned ulcers of the leg, by Goupil, &c. Chilblains are also considerably benefited by frictions, with creosote ointment or water. M. Regnart, of Paris, among many other patients, had the good fortune to relieve the gifted Broussais from an excruciating tooth-ache, by the free application of concentrated creosote to the carious tooth; we cannot wonder that the worthy professor should be an advocate of the doctrine of 'irritation.'

As this application of creosote may be of more extensive and familiar use than many others, it may be well to inquire how it acts as a sedative in this instance. When the teeth are painful it is almost always because the nervous pulp near to the root is exposed to the contact of the air. If in this circumstance a few drops of undiluted creosote are applied, a fierce increase of pain is the first effect, then a total cessasion of it: in this, either the nervous pulp is destroyed as by some caustic: or the creosote, by coagulating the albumen of the fluids that are always flowing from the caries, forms an albuminous layer that defends the pulp from the air; or lastly it acts as a powerful stimulant, causing the inflamed vessels of the pulp to contract and expel the load of blood by which they are oppressed. In any case the pain is apt to return, and this fact is only explicable by one of the two latter suppositions; for so long as the irritating cause, carious bone, remains, so long are the vessels of the pulp liable to relapse into their former congestion.

Creosote has been employed by the French physicians in pulmonary phthisis, but from all that I have read on the subject, the alleged successful cases are strained, and should not be recorded as such. It has not been more successfully used in several cases of chronic bronchitis by inhalation.

British practitioners have not as yet essayed the effects of creosote, and indeed this is too often the case with regard to new remedies. My friend, Dr. Copland, however, is an exception to this rule; he tells me he has employed it in cachectic affections as a tonic, and also in dropsical cases, where it has proved diuretic. In two cases of diabetes, he considers that he was not allowed to make a fair trial of it. The dose he gives is generally from 1 to 8 minims three or four times a day, combined with powdered liquorice root, into pills. In porrigo favosa, he has used a lotion of a saturated solution of creosote with good effect.

My own experience of the effects of creosote is as yet confined to cases of scrofulous ulcers of the leg, tooth-ache, lumbago, and aphthæ. In the first case, of ulcers, I premised a seton in the arm, and the rapid desiccation of the ulcers caused by the creosote had no ill consequence on the brain or any other viscus. In tooth-ache I have verified the reports above alluded to. In the case of rheumatism I found the remedy at first produce distressing nausea, but the warm and copious sweat that ensued soon compensated for that symptom, and effectually removed the rheumatic pain; copious diuresis was also one of its effects.

In a case of extensive aphthous ulceration of the mouth occurring in an adult, I employed the following wash with the greatest advantage; the sloughs came away after the second time of washing, and the depressions in the mucous membrane filled up with astonishing rapidity; several of the ulcerated surfaces were as large as half of a sixpence.

| | |
|---|---|
| Creosote . . . . . . . . . . . | ½ a drachm |
| Gum Arabic mucilage, | 1½ ounce |
| Camphor Mixture . . . | 10½ ounces |
| Mix. | |

To wash the mouth every two hours.

### *Mode of administering Creosote.*

M. Reichenbach says, that his observations demonstrate that in the cure of certain ulcers, tetters and wounds, creosote water was sufficient. But it should be remembered that water does not dissolve more than about 1-80th of creosote—a proportion that will be found most inefficient in the generality of obstinate cases of ulceration. In such instances the employment of pure creosote becomes necessary.

The application of concentrated creosote to ulcers, causes, in the first instance, more or less of inflammation, which, however, quickly subsides; as soon as this inflammation appears, the remedy should be discontinued for a few days, and the wounds allowed to remain quiet. The application is then renewed with similar consequences; and this is repeated until the bad condition of the ulcer is changed, that is, until the greenish pus becomes white, the blue or white flesh becomes red, and firm granulations fill up the solution of continuity. Creosote should therefore be employed more freely at the commencement than at the close of the treatment of these cases. When the ulcer has taken on the appearance above described, it will suffice to dress it with the creosote ointment, or water, or even desist altogether from its use, and introduce other desiccating remedies.

The best mode of applying it, is by means of a camel-hair brush to paint the surface of the sore; or from five to a dozen drops may be placed on the surface of a poultice, and this applied over the diseased point.

When used to stop external hemorrhages, it may be poured by drops into the wound; but in these cases it seems to act with more certainty if imbibed by cotton or lint, and thus applied.

To form a lotion for the employment of frictions, from two to eight drops are added to each ounce of distilled water. Creosote ointment is made from ten drops of that substance and one ounce of lard: it may be used either to dress ulcers or to rub into the sound substance.

The internal administration is either in draught or pills; the former being composed of one or two drops of creosote dissolved in camphor mixture; the latter of the same quantity and some absorbent powder with mucilage. This dose may be repeated three or four times a day, and may gradually be increased to eight drops.

The inhalation of creosote is effected, first, by steeping paper in it and placing this in approximation with the nostrils; next, by heating the creosote in the immediate neighbourhood of the patient, so that he cannot fail to inhale the fumes: or, lastly, a portion of it may be poured into hot water in a Mudge's inhaler, and the creosoted vapour inspired in the usual manner.

It should not be continued internally for too long a period, for it is apt to produce irritation of the system and pains of the stomach and intestines. Demulcents should accompany its employment, and should occasionally replace it.

From all that I have advanced concerning the therapeutical properties of creosote, the following general conclusions may be made.

1. That creosote is beneficial in the different stages of burns.

2. It cures the majority of herpetic, furfuraceous, squamous, and crustaceous skin diseases.

3. It cicatrizes obstinate syphilitic ulcers, prevents or diminishes suppuration, and destroys the fungous growth without injuring the surrounding tissues. It also corrects the condition of cancerous ulceration.

4. In phthisis and bronchitis, though it fails to cure either, it facilitates greatly the expectoration.

5. Chronic lymphatic tumours are frequently dispersed by frictions or fomentions of creosote.

6. It is almost always successful in allaying the pain of carious teeth, but does not prevent its return nor the progress of the caries.

7. It arrests capillary hemorrhage with remarkable certainty, but fails in that from the large vessels.

8. It is an effectual remedy in atonic rheumatism, and may be tried with chance of success in cachectic disorders."*

---

OCCLUSION OF THE BILIARY DUCTS.

In several numbers of this Journal, we have noticed this portion of pathology. In the fifth volume of the Calcutta Transactions, we find a few cases of the disease in question, and which we consider to be worthy of notice.

*Case* 1.—Keating, aged 28, a slight made and delicate European, who had been employed in Bengal for several years as an Indigo-planter, came under my care on the 18th March 1828. He had been suffering from slight pyrexia, and constipation for two days; there was general tension of the belly, and enlargement of the liver, with pain in that organ, and at the right side of the neck. Leeches were repeatedly applied, and mercurial purges administered, which operated very freely; and the mouth was affected by the calomel on the morning of the 20th March. The

* "I believe that Mr. Morson, of Southampton Row, is the only manufacturer of creosote in London."

pain in the right side was moderated, but the liver remained large. Either Aloes, Castor Oil, or Senna and Salts were given daily, until the 4th April, so as to keep the bowels very free. He had then a return of occasional pain in the right hypochondrium, and at the right side of the neck; his mouth having not quite recovered from the effects of the calomel, he was ordered to take Blue Pill, Colocynth, and Aloes, every night.

On the 7th April, there was some pain in the region of the gall-bladder, in consequence of which, leeches were again applied daily, until the 11th, when the pain had nearly subsided. A slight enlargement of the spleen was now observed, and it continued progressively to increase until he died, on the 4th July.

He suffered during this period from frequent returns of fever; and latterly had a troublesome cough, with muco-purulent expectoration, and difficult respiration, from the encroachment of the spleen, on the left side of the chest; the enlargement of the liver having entirely subsided. After the spleen began to enlarge, the use of mercury was suspended, and his treatment consisted in the repeated application of leeches, and the exhibition of purgatives, with Quinine, when required by returns of fever. All the remedies usually employed in cases of tumid spleen, were tried, with careful attention to diet, but without effect.

On dissection, the liver was found diminished in size; of a livid red color, inclining to brown: its consistence was soft and doughy. Some concretions resembling yellow soap, were found in the pori biliarii. The cystic duct was obliterated, a mere membrane of extreme tenuity remaining in its place; so that we may believe this morbid change to have been of long duration. The gall-bladder was shrivelled, its coats thickened, and it contained a small quantity of dark colored thin paste, like a mixture of charcoal and oil; insufficient in quantity to distend the diminished gall-bladder, which, therefore, had the lax shrivelled appearance just noticed. There was abundance of yellow bile in the duodenum. The spleen was much enlarged, indurated, and friable in texture."

It was ascertained that this man had had ague in the preceding Jan. attended with cough and tenderness of the epigastrium, which was relieved by bleeding and purgatives. The enlargement of the spleen appears to have swelled when the liver subsided.

*Case* 2. Macan, aged 20, and delicate, arrived from England, 20th May, 1829, having then a disease of the knee-joint. While in hospital for this, pulmonary disease became developed, and abscess of the liver was formed, of which he died on the 7th of September.

On dissection, a great many organic lesions were found in the lungs, liver, mesentery and intestines. The cystic duct was completely impervious. The patient passed black or green stools for several days about the middle of August, and therefore, Mr. T. observes, the colour could not have been owing to cystic bile. The fact is that the bile changes colour, while passing along the alimentary canal, by mixture with various matters, and especially acids. Many morbid secretions, too, are considered to be bile, when they have no claim to the title.

*Case* 3. In this case, that of an intemperate female with greatly enlarged liver, the latter organ was granular, and easily lacerable—and the *hepatic* duct was found impervious, being "degenerated into a minute film of membrane." It is curious that in this case there was no jaundice. What could have become of the bile that was secreted? It is very probable that, in such cases as these, mercury would be injurious, by stimulating the liver to increased secretion, when there was no outlet for the bile.

*Case* 4. In January 1831, our author was requested to see a man who had been, for six years, addicted to drunkenness, and, for the preceding six months, had hardly ever been sober. He was 44 years of age—pale, and emaciated. He appeared to be exhausted by disease, but without any tangible or visible malady. He only complained of uneasiness in his belly, for which some colocynth and blue pill were given. It was reported that the stools were dark and glutinous. He died in eight days after this.

"On dissection, some old adhesions were found in the right side of chest. The liver enlarged and indurated; the convex surface rough and tubercular; its texture hard, granular, and tough; in color and firmness, not unlike boiled cow's udder; being of a pale yellow color, with slight pink tinge, and bleeding little when cut. The intestinal extremity of the ductus communis choledochus, obliterated for 1½ inches; gall-bladder large and lax, contained ℥ ii ss. of pale yellow fluid, like glue. The peritoneal surface of stomach and intestines pale, and void of vascularity; the interior of reddish brown color, and covered with tenaceous viscid mucus, of the same color. Root of mesocolon and mesentery very vascular, and ecchymosed in some parts."

The most remarkable thing here is the total absence of jaundice, notwithstanding the complete obliteration of the ductus communis! This is inexplicable—unlesss we suppose that no bile was secreted in the liver—a thing hardly credible.

*Case* 5. This was also a drunkard, 45 years of age, and nineteen years in India. He was brought to the hospital labouring under ardent fever, of four days' duration. He was jaundiced —belly tumid—symptoms of delirium tremens. Antiphlogistic treatment was employed, but he died in three days. On dissection, there were morbid appearances in the head, the consequences of the fever. The gall-bladder was small, flaccid, and covered by a false membrane. It contained about a spoonful of thick black bile, like tar. The cystic duct was closed throughout its whole length. The hepatic duct was large, and contained thin bile, of a pale colour. There was much of this kind of bile in the duodenum.

---

OBSERVATIONS ON THE OBSTETRIC EXTRACTOR—THE INSTRUMENT USUALLY CALLED THE MIDWIFERY LEVER. By JOHN BREEN, M.D. formerly Assistant to the Dublin Lying-in Hospital.

This paper is published in our respected cotemporary of the Sister Isle, avowedly in consequence of a paper read by Dr. Churchill, at the College of Physicians (Dublin), on Instrumental Delivery, from which was excluded all allusion to the lever. In the opening of the paper, Dr. Breen points out some numerical mistakes in Dr. Churchill's paper, and then he proceeds to a concise, but rather full history of the forceps and vectis, from the time of the Chamberlens, in the latter part of the seventeenth century, down to the present time.

It appears that, within these few years, the lever has risen in the estimation of accoucheurs. Dr. Blundell considers it a safer instrument in the hands of general practitioners than the forceps.

"I have been (says Dr. Breen) in the habit of using the obstetric extractor for twenty-nine years, not as a lever of the first class, but as an extractor in the way mentioned by Dease. The instrument I have always used is that usually called Lowder's lever. When the hinge with which it is provided, for the purpose of being more conveniently carried in the pocket, is fastened by the pivot, this extractor is twelve inches in length. The handle, which is steel on its inner part, and partly ebony on its outer, is five and a half inches long. The blade, before being curved, was rather more than seven and a half inches. This, I believe, gives as great a degree of curvature as is consistent with the facility of introduction, and affords considerable extracting power. It is what artists call fenestrated at the extremity of the blade, or it has an oval aperture there, for the purpose of giving a greater number of points of contact with little pressure. The instrument has its greatest breadth, one inch three quarters, at the widest part of this oval. The description need not be more minute, as surgical instrument makers are sufficiently familiar with the construction of Lowder's lever."

Again :—

"To justify the use of the obstetric extractor, it is necessary that the pelvis be not materially deformed, that the os uteri be fully dilated, or very nearly so, and that the os externum be in a yielding state. I have proved in a former dissertation, that unless accidental occurrences, such as rupture of the uterus, puerperal convulsions, hæmorrhage, or other rare contingencies take place, we may wait with safety thirty hours for the condition of the parts above described, and use appropriate means during the interval to promote such a condition. I would here observe, that though the safety of delaying instrumental aid for thirty hours be proved as a general proposition, it by no means follows, that the obstetrician should always put off affording extraordinary aid for that period."

We are tempted to make another, and rather a long extract, on the application of the lever in actual labour.

"In the most frequent vertex presentation, where the labour has made some progress, and where the os uteri is dilated entirely, or nearly so, the posterior fontanelle is found to the left of the pelvis, near the left acetabulum, the sagittal suture traversing the pelvis obliquely, the anterior fontanelle generally higher than the posterior, in the majority of instances corresponding to the right sacro-iliac syncondrosis, shewing the chin to be bent on the chest. In this case the right parietal bone is the part of the fœtal cranium which is lower than any other. The head is propelled downwards with a rotatory motion from left to right, and generally expelled before the base of the occipital bone is moved completely round to the arch of the pubis. This last statement is not quite in agreement with the more general description of authors, but I am satisfied future and attentive observations will confirm Professor Naegle's views on this point. In this most favourable presentation the uterine action is occasionally for hours exerted in vain from causes which we are frequently unable to account for. Much delay may excite fears for the safety of the child, and lay the foundation of a tendency to inflammation in some of the soft structures of the mother, indicated by some or several of the following symptoms: increased frequency and fulness of pulse; tongue loaded in its centre; secretion of urine diminished, and becoming higher in colour, sometimes requiring to be drawn off by the catheter; countenance assuming an anxious aspect; stomach irritable; general increase of restlessness. This state of things, not an imaginary fiction, but one which I have witnessed, allows me an opportunity of describing how the obstetric extractor should be applied under such circumstances. The instrument can be passed with the greatest ease, as the patient lies on her left side, in the direction of the hollow of the sacrum, more to the right of the pelvis, than to the left, it is to be carried forward under the right ischium, and cautiously passed until the extremity of the instrument reaches the base of the cranium, somewhere near, but beyond the mastoid process of the temporal bone. Were I giving a lecture to mere tyros, I should be under the necessity of laying down the process by which the relative direction of the occiput to the pelvis might be ascertained. Writing for practitioners, I take for granted, that they possess this very necessary preliminary information. Yet it may be useful to recommend the operator, before introducing the extractor, for fear of mistake, to reconsider the view he has taken of the position of the fœtal head, on the accuracy of which his success must depend. Should he have formed his judgment before the cranium has escaped from the cervix uteri, the contraction of this organ on the easily compressible anterior fontanelle, will frequently lead the hasty or less experienced to mistake this for the posterior. This contraction affords an explanation of the hitherto prevailing error, as to the great frequency of the occurrence of the first position of Baudelocque, particularly as compared with his fourth. The influence of this sphincter-like action is well illustrated by the certainty with which we sometimes ascertain the anterior fontanelle, when the membranes, rigid and unbroken, contain a large quantity of liquor amnii, the interposed fluid taking off the pressure from the cranial bones. Should he, as it were, wish to check his opinion, founded on the situation of one of the principal fontanelles, by finding the direction of the helix of the ear, which is always towards the occiput, let him recollect, that if, in examining, his finger change the direction of the helix towards the forehead, the head is always sufficiently pressed to the pubis to retain that appendage of the cranium in the irregular situation which the search for its direction may have placed it in. When called on after violent labour has lasted many hours, and that there is considerable cranial tumour, this precaution will be found useful, and as far as I know, it, as well as the *cause* of the common mistake of the great frequency of the first position, is now first communicated to the profession.

The operator should now gently use some force, to ascertain if he have a purchase. He must then during a pain draw downwards, and in a direction from left to right, to bring the occiput from the left of the pelvis towards the symphisis pubis. By this mode of action the head can generally be brought to commence the distending of the perinæum, and the remainder of the process be left to nature. This power of *readily* withdrawing the extractor before the final expulsion of the head, is a most incalculable advantage; I am satisfied the injury done to the perinæum, when the forceps are used, and which is so much dreaded by all experienced obstetricians, is universally produced by the rapidity of the expulsion of the head after the chin departs from the chest, and the difficulty, or often impossibility, of withdrawing the forceps with sufficient quickness to allow our undivided attention to be applied to the care of the perinæum. On this subject, the opinion of Madame Lachapelle, whose opportunities of observation were unequalled by any writer of the present day, is so much to the point, that I feel pleased in having her authority to support my views. She says, vol. i. p. 46:—'In the last period of labour, it (the forceps) will determine almost inevitably this rupture (that of the perinæum), if care be not taken to withdraw it some moments before the complete termination." In this position of the head I have sometimes found, that the extractor, applied as I have just pointed out, did not seem to act, as it were, favourably on the part on which it was placed, and it has struck me, that the expelling power had forced the right parietal bone too low, in proportion to the remainder of the head, and that the power of the uterus still acting on this part was acting in vain. Whether my view be correct or not, I have certainly succeeded in such cases by removing the extractor to the opposite side, and with a very slight degree of force applied to the head, giving that part a direction downwards, and from left to right, effected the delivery. When the head begins to distend the perinæum, I always withdraw the extractor and leave the expulsion to nature, by which it is generally accomplished; very rarely in such a case the pains again become unavailing, and require the renewed application of external aid. The head is now so low, that a very slight extrinsic force added to the uterine action, however slight this may be, will disengage it. I never met an instance where the instrument passed obliquely across the bottom of the pelvis, the handle to the left, the other extremity towards the opposite ischium, in which a very slight exertion of power, directed to move the forehead from the hollow of the sacrum, did not disengage the head."

We think our obstetric brethren, a pretty numerous class of readers, will not grudge the time spent in perusal of these extracts from the paper of an experienced practitioner.

Observations on Lepra and Psoriasis. By C. W. Pennock, M.D.*

Great attention has been directed for some years past to cutaneous diseases. Practitioners of every grade are becoming aware of the necessity of studying their various characters, and discriminating their several kinds. Yet still much obscurity prevails, even among scientific men, with respect to their distinctions, and no little confusion undoubtedly exists in the minds of too many members of the profession with reference to their treatment. As a general rule we may say that at present there is too great a leaning to the employment of specifics, and too little confidence in the application of those general principles of treatment, which probably exert almost as much influence on the diseases of the skin, or at least on the states of system that produce them, as on other maladies, the general management of which is understood.

The publication of cases of affections of the skin in periodical publications has not been sufficiently adopted. Cases published in this manner become more familiar, and bring the characters of disease and its treatment more home to the mass of professional men, than the formal contents of elaborate works can ever do. Experience obtained by the observation of individual cases, and perhaps the best substitute for such experience, or rather for the mode in which it is acquired, is the record of cases of a similar description. We purpose, therefore, to lay before our readers such facts and observations as appear from time to time in monographs or in other journals, facts and observations which may seem adapted to increase the general amount of information on the subject of cutaneous maladies.

The paper before us contains the result of the observations of Dr. Pennock, in the Hôpital St. Louis. At that hospital the student enjoys the opportunity of listening to the clever fancies of Alibert, and the valuable lessons of Biett. He enjoys, also, another and a far superior advantage, the power of watching a great number of cases, of studying Nature on a splendid scale. Let us now introduce our author, and his facts to the notice of our readers.

The paper is confined to the elucidation of the characters and treatment of lepra and psoriasis. In order that no misconception should occur, we should explain the sense in which those terms are used.

" Lepra, in accordance with the definition of Willan, signifies a cutaneous squamous affection, characterised by round patches, raised on the borders, depressed in the centre, from which exfoliations of diseased cuticle take place, independently of any vesicular or pustular formation.

This disease makes its appearance by the formation of slight elevations resembling enlarged papillæ of the skin, which are firm and solid, and which are covered with thin, dry scales; small circular and raised patches of diseased surface form; the central portion soon becomes healthy, leaving raised circles of morbid structure of the skin."

" Psoriasis bears so striking an analogy to lepra, that both have been considered as being essentially the same disease. In each the commencement is by small and hardened elevations, covered by thin, dry scales; the causes producing them are the same, and the only important differences existing between them, are the varieties of figure. In confirmation of the truth of this position, it may be observed, that spots of lepra corresponding with the description of Willan, are frequently seen intermixed with those of the irregular patches of psoriasis."

Eight cases are detailed, and remarks on the general and distinctive features of psoriasis and lepra are appended to them. The majority are instances of psoriasis, but we shall take those of lepra first.

Case 1. *Lepra Vulgaris—cured by the Homœopathic Treatment—Relapse at the end of five months.*

* Amer. Journ. of Med. Sciences. February, 1835.

A locksmith, æt. 21, entered l'Hôpital St. Louis, in March, 1833. The legs, arms, head, presented an eruption in the form of small scales on a red base, which was slightly raised above the level of the skin; these scales were dry, and of a white, shining, micaceous appearance. The elevations were circular, and varied in diameter from two lines to half an inch; they were equally raised in the centre, as at the circumference, and touched each other at their outer border. Their appearance was that of psoriasis guttata. They covered the back, breast, and the external part of the superior and inferior members. The disease had commenced in March 1832. It then resembled the present affection; in three months it was removed by sarsaparilla, alkaline and sulphur baths, and the internal use of pills of some sort. In July 1832, the disease returned and continued till the time of his admission.

It was now treated homœopathically. The patient was ordered a solution containing a millionth part of a grain of arseniate of potash, which was prepared in the following manner:—A grain of the arseniate of potash was dissolved in an ounce of water, which contains six hundred drops; one of these drops containing one six-hundreth of a grain, was put into an ounce of distilled water, of which one drop then contained one thirty-six hundredth of a grain of the salt, and thus the dilution was proceeded in, until the minute dose of a millionth part of a grain was obtained.

The patient was interrogated each day respecting his feelings, the state of the secretions, &c.; in a word, as regards his general health. No perceptible effect was produced, yet notwithstanding this, the patches became smaller, the desquamation ceased, and on the places which had been affected by the disease, circles were seen, where the skin was grayer than that of the healthy parts, but without being either raised or depressed; these spots rapidly became paler, and the patient left the hospital on the 3d of June, completely cured. He had taken during his residence there, one eight-thousandth part of a grain of arseniate of potash."

On the 2nd December 1833, the patient returned with small circular spots of psoriasis on the scalp and back.

The preceding case may be made a present to the homœopathists. Those who are disposed to believe that one eight-thousandth part of a grain of arseniate of potass removed the patches of lepra, will, no doubt, be gratified with the issue of the case. The incredulous will display their usual scepticism, and hold in contempt the folly or the knavery of the disciples of Hahnemann.

We suppose that Nature may safely be allowed the credit of doing as much in this instance as the eight-thousandth part of the grain of arseniate of potass. But we will not presume to insist on this rational opinion, and we leave to the homœopathists the laurel and the crown. Dr. Pennock observes that the lepra vulgaris in this case commenced in the form of psoriasis guttata, and that such has been the mode in which all the cases of lepra vulgaris he has witnessed did commence. Ordinarily, the psoriasis diffusa is seen intermediate between the psoriasis guttata and lepra, but here the transition from the psoriasis guttata to lepra was direct.

Case 2. *Psoriasis Guttata and Diffusa, with Lepra Vulgaris—treated by Frictions with Ioduret of Ammonia.*

A weaver, æt. 27, admitted in April 1832. The disease had commenced in November 1827, in the form of isolated pustules. It had been at various times and under the influence of various remedies temporarily benefited. On his admission his condition was as follows.

"The arms and legs of the patient present,

*First.* Spots, some of which are as large as the hand; others the size of a ten cent piece, red, raised, and covered with scales, which are dry on their internal and external surfaces, and of a pearly whiteness.

*Secondly.* Circles from one to two inches in diameter, of which the borders are elevated and covered with scales, whilst the central portions are healthy, and on a level with the sound skin. These are situated principally on the abdomen.

*Thirdly.* Other spots of the size of a small pea, which are slightly raised above the skin, are of a bright red colour, which disappears upon pressure, and are covered with minute scales having the characters of those first mentioned; these elevations are the seat of violent itching. In the course of two months these spots become much enlarged, and form patches an inch in diameter, the skin in the centre of which is without scales, smooth, and healthy; the outer border remaining elevated, and covered with small, dry scales."

For two months, pills of the sulphuret of antimony were exhibited; but were discontinued in consequence of the violent gastric pains they occasioned. The tincture of cantharides was next administered without producing any sensible change. On the 1st of August, he quitted the hospital, and on the 6th of September he returned. The "Fowler's solution" was now given for a month, without service. On the 2nd of October, M. Gibert directed the employment of frictions with the ointment of the ioduret of ammonia, ℥ss. morning and evening.* These frictions were continued for twenty-nine days, when the patient took eight alkaline baths. The result of this treatment was, we are told highly satisfactory, all the spots disappearing, and their places being indicated by a slight red colour. By the 5th of December, however, the disease had re-appeared on all sides, in spite of the resumption of the frictions. He now gave up all treatment until the ensuing Winter.

Dr. Pennock remarks that, in this case, lepra and two forms of psoriasis were seen on the body of the same individual, confirming, he thinks, the opinion that they are essentially the same disease. That they are essentially of the same class we admit, depending, probably, on similar causes, and originating in a similar state of constitution. Thus the syphilitic tubercle, lepra, and psoriasis are evidently connected with each other, depend on the same state of constitution, and require the same kind of treatment. But they are not therefore the same disease, because they present characters differing in some respects, and run a course which also exhibits some distinctive features. Such is the case with common lepra and psoriasis, and we conceive that, though in many particulars analogous, they are far from being actually identical.

* "The formula for the preparation of this ointment varies in proportions of the ioduret, from ℈j. to ʒj. to an ounce of adeps. The weaker preparation being used when the disease is recent, and the stronger when it is chronic. As the ioduret decomposes by exposure to the air, the ointment should be kept in stopped bottles."

Case 3. *Psoriasis Inveterata — treatment by the Sulphuret of Antimony, subsequently by the Ointment of the Ioduret of Ammonia.*

A shoemaker, æt. 40, was first affected with psoriasis, which commenced behind the ears, in the Spring of 1809. From that time till October 1832, the disease had made progress, but not uniformly; for it had been temporarily benefited upon more than one occasion.

On the 22nd October 1832, he was received into the Hôpital St. Louis. The treatment at first consisted in the infusion of succory; subsequently that of hops, conjoined with pills of the sulphuret of antimony. Of the latter, commencing with one pill a day, and gradually increasing the dose, he took sixty-three pills in thirty-four days. In the middle of December, we find the following specified as the condition of the patient. The entire scalp is covered by small yellowish scales, which extend over the forehead and temples. On the nape of the neck, and on the back, are twenty patches of various sizes, elevated, red, of a circular form, and covered with light, dry, thin scales. On the breast are two large patches; one of which was cauterized a month ago by the pernitrate of mercury, but the scales have again formed upon the cicatrized surface. Five patches are seen on the abdomen: on the forearms several eruptions originally distinct from each other have united, and envelop the limb in its entire extent.

The skin covering these surfaces is raised, red, traversed by numerous transverse furrows, which are again crossed by superficial depressions running longitudinally. From these intersections result small squares, covered by dry, white, thick scales, which are very different in appearance from those of psoriasis diffusa. There were patches on the lower extremities, but their elevation was less than on the upper.

On the 25th of February, no marked amelioration had occurred. The antimonial pills were now suspended, and frictions of the ointment of emetic tartar substituted. The effect of this was to produce much inflammation and pustules, and give the psoriasis a resemblance to impetigo. In April, the tartar-emetic ointment was abandoned, and the tincture of cantharides given internally. It occasioned nausea and diarrhœa, and was abandoned also. On the 26th of October, the patient was directed to make use of frictions with the ointment of the ioduret of ammonia, ℥ss. every day. On the 28th of December, the last time when Dr. Pennock was able to inspect and to report upon the case, it appeared much improved. The patches, says the report, are very slightly elevated; they are of a pale red colour, which disappears under the pressure of the finger, and the cracks and furrows formerly observed, have almost entirely disappeared; a slight formation of thin, small and dry scales is still seen. Interspersed amongst these spots are portions of skin, which is not rough, hard or dry, but which has become soft and pliant; in a word, perfectly healthy.

The patches of psoriasis of the body have undergone the same alterations as those of the arms; they are less numerous, very slightly elevated, and the desquamation is altogether furfuraceous. None of these spots present the rough cracked appearance which was formerly seen upon their surfaces.

In the next case, (the 4th) related by our author, lepra vulgaris was apparently removed by the supervention of variola. On the subsidence of the latter, the lepra also faded, and ultimately disappeared. In the fifth case, a complication of variola and psoriasis diffusa proved fatal, and afforded an opportunity for examination after death.

*Case.* The patient, a shoemaker, æt. 26, entered the hospital on the 19th December, 1831, affected with psoriasis diffusa, which had commenced in the September previous. On the 30th he was seized with chills followed by pyrexia, and on the 2nd of January, small-pox made its appearance. The eruption became confluent. On the 6th, there was intense angina, with inclination to coma. *V. S. ad* ℥viij. On the 7th, the coma was diminished, but the left eye was much inflamed. On the 8th, delirium with nausea and vomiting—pulse frequent and weak—deglutition almost impossible. The prostration increased, though the other symptoms displayed some variation. On the 10th, he died.

*Dissection, 24 hours after death.* "*Cranium.*—The cerebral veins were engorged with blood; the sinus of the dura mater, as well as those of the brain, were empty. The medullary substance of this organ firm, and clotted with red points of blood.

*Face.*—Conjunctiva of left eye very red and inflamed; olfactory membrane covered with pustules.

*Respiratory organs.*—Mucous membrane of the epiglottis of a deep red colour, very small pustules are seen in the glottis. The mucous mem-membrane of the larynx and trachea is much injected and thickened; that of the bronchi of a scarlet redness to their most minute ramifications. *Lungs.*—Crepitant in some portions, but generally engorged with black blood, as in death from asphyxia. *Heart.*—Natural.

*Digestive apparatus.*—Tongue covered on the edges with eight or ten pustules; the amygdalæ swollen. Pharynx red; the superior portion covered with large variolous pustules; no preternatural redness of the œsophagus; its glands are of the size of a millet-seed; on some parts its epidermis is detached. *Stomach.*—Mucous membrane of a brownish-red colour; its consistence normal. *Duodenum.*—Mucous membrane of a pale red, not softened. The follicles of Brunner were numerous in the upper portion of the intestine. *Peyer's glands* were not developed. Towards the last fourth of the small intestines, Brunner's glands were much enlarged. The *ileum* was of a deep red near its junction with the cœcum. In this last intestine the follicles were of their natural size. The mucous membrane of the colon was red for the space of a foot; the follicles of the rectum were visible, but not as much enlarged as those of the small intestines.

*Skin.*—The pustules of variola, as well as the patches of psoriasis, are not so elevated as during life. Upon examination of the pustules of the small-pox, the cutis vera was found perfectly healthy; the superposed layers described by CUTUGNO were very evident, and coloured by blood from the capillary blood-vessels of the skin. In the patches of psoriasis, the derma was also found in a normal condition, but the rete-mucosum was exceedingly red, and the sanguineous infiltration much more copious than in the variola, giving a red tinge to two false membranes, which were separated by bloody pus."

Dr. Pennock observes, in reference to the preceding interesting dissection, that it exemplified the fact of inflammation of the rete-mucosum as an attendant upon, and probable cause of psoriasis. M. Rayer states, that this is always the pathological state of that portion of the skin in this disease. Plumbe ascribes it "to a chronic inflammation of the vessels secreting the cuticle, producing morbid growth of this structure, and generally dependent on debility of the system."

The chief peculiarity of the sixth case related by Dr. Pennock, is the circumstance that, in its progress, it displayed the sinuous patches of psoriasis gyrata. M. Biett had seen four instances only of this rare variety. Pills of the sulphuret of antimony, the arseniate of ammonia, the tincture of cantharides, the ointment of the ioduret of ammonia, &c. proved perfectly inefficacious.

CASE 7. *Psoriasis Inveterata—benefit from the use of Pearson's Solution.*

A cooper, æt. 57, first became affected with psoriasis in 1823 or 1824. It appeared upon the scrotum in the commencement. On the 10th of October, he entered the Hôpital St. Louis.

"The external two-thirds of the right arm are covered with continued patches, of which the base is a pale red, slightly raised above the skin, the surfaces of which are covered by large, thin, dry scales, of a pearly whiteness, and are separated by longitudinal ridges. Other elevations are observed, from which the scales have been torn by the nails of the patient, leaving the red surface exposed. Instead of the borders ending abruptly by round edges, as psoriasis usually does, this case presents a different appearance. The diseased surfaces gradually lose their elevation; the red colour of their base changes by insensible degrees to a light brown, and this again into the natural colour of the healthy skin.

The second remarkable circumstance is, that there is no trace of other spots or patches of psoriasis, either near the large one on the arm, or near those of the body.

An eruption like that on the arm surrounds the neck. The scrotum is hanging, has a red dry appearance, and is covered with scales of a yellowish colour; so that without the psoriasis of the body, it would be taken for an eczema; frictions having modified the appearance of the scales.

The entire scalp is affected, and the scales which are there of a more brilliant white than on the body; they are very small, and resembling the furfuraceous appearance of pityriasis; on the anterior part of the cranium the red base and elevations of psoriasis are scarcely visible: on the back part of the neck, on the contrary, the rounded edges and raised border are covered with tolerably large scales. It would seem that the neck is covered by psoriasis, whilst on the anterior part of the head an eruption intermediate between pityriasis, rupia, and psoriasis is presented.

*Treatment.*—In thirty days the patient took twenty-four and a half drachms of *Pearson's solution,* during which time his usual drink was *an infusion of the saponaria officinalis,* under which treatment the affection was mitigated."

This " mitigation" augmented to convalescence, yet the patient was certainly not cured when the report concluded.

CASE 8. *Cutaneous Affection intermediate between Psoriasis and Eczema.*

We notice this case because it displays a condition intermediate between psoriasis and eczema, a condition which we do not think extremely rare as we have lately witnessed two examples of it. But we will mention the particulars of the case of Dr. Pennock.

A labourer, æt. 57, who had lived chiefly on bread and cheese and brandy, thought that he observed the complaint in August, 1832. It commenced on the right elbow, from which it extended to the remainder of the body. In March, 1833, he was received into the hospital. His state was then as follows, and we pray the attention of our readers to the particulars.

" On the back are observed patches of diseased skin, varying from two lines to an inch in diameter, and of an elliptical form. These portions are covered with scales, which being detached, the skin is seen of a pale red colour, in most instances not raised above the sound skin, and in others very slightly elevated. The scales on their external face are white, micaceous and reticulated; internally they are slightly yellow. They are evidently formed of the epidermis, and appear to be composed of two laminæ.

Towards the back of the neck, and on the shoulders the patches unite, and form a continuous series; the skin composing them is red, slightly raised above the level, humid from serous effusion, and covered with dry, thin, yellow scales. The same appearances are presented on the abdomen as on the back.

On the arms, particularly on the internal parts, are seen spots, (plaques,) which are raised the fourth of a line above the sound skin, of a red colour, and from two lines to an inch in diameter, and covering the elbow joint; these spots are divided longitudinally by furrows, and the desquamation consists of crusts which are yellow, of various forms and dimensions.

On the scrotum, and on all the inner surfaces of the thighs, the skin is red; these parts are covered with thin yellow scales, some large, others small, varying from a line to half an inch in diameter. The internal face of these scales is generally humid, and on the whole of the inner part of the right leg is an abundance of serous exudation. On the legs the scales do not present the white micaceous aspect that is seen on those of other parts of the body.

On the scalp, the eruption has the appearance of psoriasis."

As Dr. Pennock was unable to follow up the case, we need not enter on the treatment. In fact, the main interest is connected with the complication of the characters of psoriasis and eczema. We mentioned that we have lately witnessed two instances of this description. The last was in the person of a gentleman who applied to us a few days ago. He had had the eruption for three years, and it had commenced in India. We need not mention the particulars of the eruption, as the case of Dr. Pennock will furnish the essential features of both. It was, however, extensive and distressing.

One circumstance was common to all the cases we have seen, a tendency to occasional exacerbations and remissions. The exacerbations were always ushered in with fever, but this after a short time subsided. The general pyrexia was always attended with aggravation of the local inflammation, and the latter generally shewed a disposition to subside, in proportion as the former declined or disappeared. This circumstance, however, is observed in many cutaneous affections, and particularly, perhaps in psoriasis and eczema. We think that it deserves the especial attention of the surgeon or physician in directing the management of those affections. The remedies that are applicable to the inflammatory stage are certainly not required when the inflammation has subsided. In the inflammatory stage, mercurials and active purgatives, in some instances even venesection, salines, and antiphlogistic regimen, are the natural and indeed the most advantageous remedies. But after the inflammatory stage has passed away, sarsaparilla and the liquor potassæ, the liquor arsenicalis, and medicines of that class are indicated. Much, we think, depends on watching the state of the eruption, and on not indiscriminately using tonics or specific remedies, no matter whether there be fever or not—no matter whether local inflammation is or is not in existence.

The resemblances and distinctions of psoriasis and eczema are briefly, but sufficiently pointed out by Dr. Pennock. The points of resemblance, he observes, are as follow:—

"*First*. The epidermic secretions.

*Secondly*. The slow progress and chronic character which each may present.

*Thirdly*. The red coloration of the skin.

*Fourthly*. The intense itching.

The differences may be thus shown.

*First*. The elevation of the derma. (*Psoriasis.*)

*Secondly*. Vesicles at the commencement. (*Eczema.*)

*Thirdly*. Serous effusion, accompanied by the epidermic secretion. (*Eczema.*)

*Lastly*. The skin smooth and shining after the cure. (*Eczema*)."

We must now indulge in a long quotation, containing, as it does, a summary of the facts in the preceding cases, and of our author's observations in the St. Louis' Hospital.

"Upon an analysis" he says, "of the preceding cases, as well as some others in the writer's possession, it is observed, that the disease first appeared on the superior and inferior extremities, especially in the vicinity of the articulations, in seven out of ten instances; on the head twice, and once on the scrotum. In every case the disease commenced with the sensation of burning and itching, and the primitive appearance was that of enlarged papillæ, which were soon covered by the formation and exfoliation of minute and dry scales. The papular appearance increased, assumed a form more or less circular, the surface of which did not manifest a central portion of sound skin previous to having been covered by squammæ. The primitive appearance of lepra and the varieties of psoriasis were the same, and in two instances, patches of the former were intermixed with those of the latter; these facts, coinciding with the observations of pathologists of other countries, warrant the opinion, that the difference of the disease is merely in the form, and that essentially they are but varieties of the same affection.

In one-fifth of the cases, one of the parents of the patients had had a sqamous affection. No facts were presented which induced the idea, that the disease had been the result of contagion. The observations having been confined to persons in the poorer classes of society, no comparison could be instituted between them, and those surrounded by more comforts. Most of the patients were persons whose general health, previous to the disease, had been good.

One of the individuals attributed the origin of the disease to domestic chagrin. It is certainly remarkable, that a moral cause should produce such an effect upon the skin, but similar cases have been frequently remarked. Plumbe relates two highly interesting cases of that character. He remarks, that 'the class of persons who appear to be most subject to it, (lepra,) are those whose minds are anxiously occupied by the cares of business or study, or who are accustomed to bodily exertion beyond what their strength enables them to bear.'

Respecting the pathology of these affections, writers are far from being unanimous in their opinions. Alibert observes with great truth, 'Ce qui deconcerte l'observateur lorsqu'il est a la recherche des causes qui influent sur le développement de l'herpès, c'est de voir, ce genre d'affection se manifester chez des sujets qui jouissent, au moins en apparence, d'une santé parfaite.' Plumbe thinks that the vessels which secrete the cuticle, are the seat of chronic inflammation, which renders the production of the epidermis more abundant, and causes the exfoliation of the scales. This hypothesis is imperfect, as it does not account for the circular form which the patches of lepra present. M. Rayer attributes the diseased action of the cuticle to the inflammation of the rete-mucosum. In case fifth of the preceding series, this condition of the mucous tissue was observed; how far it had been influenced by the existence of variola, the writer is unable to determine."

Dr. Pennock does not attempt the diagnosis between psoriasis and lepra, because, as has been already mentioned, he considers them as varieties of the same affection. The presence of dry scales, he remarks, is the general distinctive character of psoriasis. But sometimes, the primitive exfoliation is superseded, or attended by serous or sero-purulent appearances; in this case the presence of vesicles or pustules may be detected on examination, and the scales are not dry, gray, and friable, but are large, soft, and humid concretions of the effused fluids.

"Chronic eczema in some instances presents appearances very similar to psoriasis; this is particularly the case when it affects the head. In this case, although the scales may be dry, yet upon examination immediately behind the ears the surface will be found moist, and vesicles are occasionally seen in the vicinity. Psoriasis of the scalp may be distinguished from pityriasis by the thickness of its scales, and by the solid papular indurations, which are more or less prominent."

On the distinctions between common psoriasis and lepra, and certain syphilitic eruptions we will not enter here. A paragraph would be perfectly inadequate to point them out, and we have not space for more extended remarks. We insert our author's remarks upon the treatment, which probably comprise the greater part of what is known with respect to the management of these most obstinate eruptions.

"Pathologists differing respecting the seat of the disease, present views equally adverse in regard to the treatment. Rayer founding his opinion on the idea, that an inflammation of the rete-mucosum is the pathological state of the skin, recommends venesection, and the application of leeches to the diseased parts, upon their first appearance. Plumbe, on the contrary, regarding this affection as the result of debility, directs his entire attention "to the restoration of the strength of the patient to its original standard, not simply before the cutaneous disease appeared, but even before those habits or pursuits were adopted, which for years may have preceded it." Hence he urges the importance of placing the patient under circumstances favourable to the invigoration of the general health. It would seem impossible to lay down any positive and unvarying plan of treatment, occurring as the disease does, in the poor, enfeebled by every variety of privation, and in the rich, surrounded by every comfort and luxury; in fact, the practitioner must be directed by his own discrimination.

The practice of M. Biett is, if the patient be young and vigorous, the skin inflamed and red, the pulse full and active, to have recourse to venesection, simple baths, diluent drinks, rigid diet, and rest. He disapproves of the application of leeches as never producing any beneficial effects. In those cases where the patient is old, enfeebled by disease or insufficient nourishment, and where there is not evidence of much inflammatory action, tonics should be administered. At St. Louis, where the class of patients are those who have been subjected to much privation and distress, the practice last mentioned is resorted to, until the state of the system is such as to support active treatment. The external applications in use at that hospital, and which have been attended with useful results, are the various preparations of iodine, combined either with sulphur, (grs. xv. to grs. xx. of the proto-ioduret to ℥j. of axunge;) or the proto-ioduret of ammonia combined as mentioned in the first part of this essay. Whilst the patient is on the use of a bitter infusion, (generally that of hops,) frictions with these ointments are made morning and night on the patches of the eruption. The result with the ointment of the ioduret of ammonia has been previously stated; that with the ioduret of sulphur is such as to warrant the continuance of its use. In a case of psoriasis diffusa, which I have recently had under my charge, the result of the treatment with friction with the ointment of the ioduret of mercury has been very satisfactory. M. Alibert used with much success the ointment of the white precipitate of mercury. Plumbe recommends in strong terms the following external application: ℞. Hydrarg. subm.; Plumb. superacet. āā. ℥ss.; Ung. hydrarg.; nitrat.; ung. cetacei, āā. ℥ij. M.

Baths are of much use in exciting the circulation, in producing a more natural state of the skin, inducing perspiration, and by detaching the scales. They must be directed on the principles previously mentioned; using the simple, emollient, or narcotic baths when much inflammatory action exists, and the more tonic in cases of enfeebled states of the system; sulphureous, sea water, alkaline baths, and those of the preparations of iodine, are then considered as most useful.

Vapour baths, either general or local, are also useful auxiliaries in the treatment of these affections.

As regards the internal treatment, Biett places much reliance on purgatives of calomel, (either alone or combined with jalap,) when administered in the forming stage of the disease. It is found to be particularly valuable in cases of children. He counsels its administration in small, in preference to large doses, with the view of producing a slow, rather than a sudden change in the system.

The tincture of cantharides has often produced much beneficial effect in chronic cases, or where the disease has reappeared, or where it exists in persons of enfeebled constitution. His mode of administering this preparation is from three to five drops in a tea-spoonful of sweetened water or ptisan, in the morning previous to eating. The state of the digestive and urinary organs are to be closely attended to, and if no epigastric pain, nausea, purging, or ardor urinæ should be induced, it may be augmented five or six drops a day, until twenty-five to thirty drops a day have been administered. In exceedingly inveterate cases of this disease, all the preceding treatment has been found to fail; in such cases the exhibition of Fowler's and Pearson's solution has succeeded. The commencing dose of Fowler's solution being three drops in some inert vehicle, and gradually augmenting until twenty-five to thirty drops per day have been administered. Pearson's solution being much weaker, may be given in the dose of a scruple, increasing to half a drachm. The preparation of the arseniate of soda is applicable to women and debilitated persons. In the administration of these remedies, the medical observer should keep constantly in view the injury which the gastro-intestinal mucous surfaces may sustain, and cease the exhibition of these preparations upon the slightest manifestation of diseased action.

M. Biett reports several cases of psoriasis inveterata, where he obtained satisfactory results by the administration of the arseniate of ammonia used in the same doses and under the same circumstances with the arseniate of soda.

In psoriasis affecting the prepuce, the application of mercurial ointment should be used. In psoriasis scrotalis, fumigation with sulphur, or cinnabar, is found very efficacious."

The plan we have found most beneficial has been the employment of antiphlogistic remedies during the febrile stage of psoriasis, or when any local inflammation exists—but when these are not present, or have passed away, the treatment must be changed for a plan of a more stimulating character. Sarsaparilla and the liquor potassæ or the liquor arsenicalis, is certainly the remedy that answers more frequently than any other, but light bitter infusions with soda, or if the patient's system be enfeebled with the volatile alkali, and gentle tonics of various kinds, are not unfrequently serviceable in these circumstances. Purgatives, especially combined with mercurials, are extremely beneficial in psoriasis. In lepra they are not so useful. In psoriasis there is generally more of inflammatory action, and a febrile state, than in common lepra. Much, however, must depend on the circumstances of the individual case, and much on the experience, the judgment, and the tact of the physician.

We think the preceding paper not unworthy of the attention of practical men.

---

### Mr. Blackley on Erythema Nodosum.

Our excellent contemporary, the Dublin Journal for July of the present year, contains a short paper on erythema nodosum from the pen of Mr. Blackley. He offers the results of his observations on the characters, and of his experience in the management of this complaint. He also presents a case circumstantially and explicitly detailed, in illustration of his treatment and opinions.

" This is a disease," he says, " of by no means frequent occurrence, insomuch, that many practitioners indeed do not recognize it when first presented to their view; it is not peculiar either to *age* or *sex*,* nor have I known it to arise in any instance from infection, on the contrary, I have witnessed many cases where other children mixed with impunity with one so affected, without exhibiting the slightest symptoms of the complaint. So far as my experience goes it never proves fatal, though it reduces the patient very considerably in strength and habit, and in adults sometimes assumes an alarming appearance on that account: it is uninfluenced by season, occurring in the depth of winter as frequently as the middle of summer, nor am I prepared to refer it to any peculiarity of habit or idiosyncrasy.* I coincide in opinion generally with Mr. Plumbe, in attributing this disease to some *' derangement of the secretions, and disordered state of those organs in which the process of chylification is carried on,* though I think the cause of it may be more *particularized.* The state of the tongue, however, and the pulse, the nature of the excretions, the proneness in children so affected to pull their lips, &c. &c., together with the good results which we observe to flow from the properly directed means adopted to remove intestinal irritation, all tend to prove, that this opinion is founded on just and rational foundations."

There are some particulars in which our experience, such as it is, militates slightly against that of Mr. Blackley. In the first place, we have found the complaint less unfrequent than it seems to have been within the circle of his observation—in the second, we have generally, indeed we have almost always seen it in young persons—in the third place, females have formed the preponderating majority of the patients, and those of a gross, sanguineous habit, with ruddy complexions conjoined with a bad circulation, have usually appeared most prone to the affection—and finally, we have certainly observed more cases in the Spring and Autumn than at other seasons of the year. But to proceed with the descriptive remarks of Mr. Blackley.

" This complaint always appears in the first instance on the *legs.* I have never known an instance to the contrary; the parts first affected are the shins, where we observe large oval patches, ' the long diameter of which, as described by Mr. Plumbe, ' is parallel with the tibia;' this has been preceded by three or four days of fever of a mild type, at the end of which time the patient perhaps complains of sore

---

* " Dr. Bateman appears to be of an opposite opinion, as he says, ' it seems to occur only in women.'—*Vide* his Synopsis of Cutaneous Diseases. Mr. Plumbe, however, very justly asserts the contrary.—See his Practical Treatise on Diseases of the Skin. Also Merriman in the edition of Underwood on Diseases of Children, edited by him."

* " I have seen it affect a gentleman, and after an interval of ten or twelve years exhibit itself in two of his children in the course of twelve months—a girl aged seven, and boy aged four years; it did not appear in the other children of his family."

legs, and on examining these we find the disease developed. A remarkable feature in this complaint is, that it is confined for the most part to the *fronts* of the legs: some spots may appear on the sides, especially over the fibula, but seldom or never on the calves, though the tendo Achillis is not always exempt: it will also exhibit itself on the arms, where the same peculiarity is to be observed, with this exception, that here the *fronts* of the arms are the *exempted* parts. In both cases, however, we may remark, that the eruption takes place over the muscles of *extension*, while those of *flexion* are not affected. The eruption exhibits all the phenomena of inflammation, redness, heat, pain, and swelling; yet in each of these a certain peculiarity is found to exist; thus, in a large blotch, the redness is first of a bright colour, not so vivid as in erysipelas, nor shining. In the course of twenty-four hours it becomes darker, and the centre now begins to approach a purple hue, which it attains in from sixteen to twenty-four hours more: in the same additional period of time it assumes a light slate, and finally an ochre colour, after which at an indefinite time, the skin regains its natural appearance. The circumference of the blotch does not keep pace in its changes with the centre, but *follows* it; thus, when the latter is purple, the former is red, appearing like a ring of inflammation surrounding the centre, and when the centre is of a yellowish hue, the circumference becomes purple, which colour is there retained after the disease has terminated; whence the appearance is presented for several days of purple rings, where large blotches previously existed. I would here observe, that it is in the *large blotches* only that these *characteristic* changes are to be traced; in the small ones they are not generally to be remarked.

The sense of *heat* is not so great as we might expect from the inflammation, neither is the *pain* when in a recumbent posture, but both are increased on the patient assuming the erect position, when a heavy, bursting, and tingling sensation is conveyed to the inflamed parts of the leg, owing of course to the gravitation of the blood; even in the *horizontal* posture *pressure* on the inflamed spots causes pain, and should be avoided. The *swelling* in each tumour appears simultaneously throughout, but *subsides* from the centre, which does not at any time feel so hard as the circumference; thus in the stage of the disease where the *centre* is slate-coloured, it is also sometimes so *soft*, as, when compared with the *circumference*, which is harder and of a red colour, to give rise to apprehension (in those unacquainted with the disease) of *sloughing* from the centre. I have never seen an instance in this disease of the skin giving way; the termination of it is *invariably* by resolution. Its duration depends entirely on the means adopted to remove its exciting cause, which it is, therefore, necessary we should perfectly understand. Agreeing as I do generally with Mr. Plumbe, in his remark before quoted, I yet think, that this disease is more *particularly* referrible to the vitiated secretions of the *liver:* the dark and fetid character of the fæces, the deep colour of the urine, depositing a heavy sediment, the loaded tongue, and headach, with frequently a sensation of heaviness in the region of the liver, would point out that viscus as being the part most probably that is primarily affected; while the happy results which flow from the use of calomel in improving the tone of this organ, proves that such a conclusion is by no means unwarranted."

We cordially coincide with Mr. Blackley in directing marked attention to the state of the secretions. We have always found them extremely depraved, and usually imperfect. The tongue has been loaded, the countenance perhaps bloated, the bowels confined, the urinary secretion commonly disordered. We fear that too many cutaneous disorders are treated empirically even by men who are otherwise scientific and judicious in their views and practice. We daily see a farrago of washes and of ointments prescribed for eczema, psoriasis, and impetigo, when smart purging, restricted regimen, and unirritating applications are likely to be attended with much more benefit.

Mr. Blackley's methodus medendi may be briefly stated:—slops for diet, till the tongue is clean, and till two or three days have passed without the appearance of any fresh spot—calomel and Dover's powder for one night, followed on succeeding nights by calomel and jalap—tonics, after the subsidence of the disease. After the termination of the complaint, the skin which has been affected remains for several days of a blue mottled appearance, resembling bruises, and frequently gives off little branny scales; the swelling also remains for a considerable time, it is most remarkable over the tibiæ and fibulæ, and remains longer in those situations than over soft parts, giving to the bones an uneven appearance, and to the touch resembling a great number of nodes of various sizes over their surface, whence of course the origin of the term, erythema *nodosum*."

We perfectly agree with Mr. Blackley in his recommendation of purgative medicines. They are absolutely indispensable. But we have sometimes found it advantageous to combine with them, in young persons with a feeble circulation, light bitters with the carbonate of ammonia. After a sharp dose or two of calomel and jalap, and perhaps senna combined with colchicum, we have prescribed the infusion of cascarilla with the spir. ammon. aromat. once or twice during the day, ordering the patient a pill of the pil. hyd. and pulv. ipec. at night, succeeded on the next morning by a draught containing the carbonate and sulphate of magnesia and the vinum colchici. Sometimes light bitters with the carbonate of soda, or the caustic alkali may be advantageously conjoined with the purgatives. We repeat, however, that the latter are the remedies on which we must principally lean.

There is nothing in the case detailed by Mr. Blackley to detain us. But he draws attention to another kind of case, less familiar to our readers, probably, than the preceding. His description of this we must give in his own words.

" A disease exists of which I have seen but few examples, which though totally differing from the preceding, might yet be confounded with it, resembling it in some of its general symptoms, but differing from it in its situation and cause, and the means indicated for its cure.

It commences by a little tumour under the skin of the back of the leg, generally the calf, feeling like a large grain of shot in that situation; without colour and without pain, unless when hardly pressed. Within about a fortnight it has increased to the size of a swan-drop, and has much the feel of an encysted tumour; the skin now begins to assume a dusky red colour, and is painful on pressure; within three weeks from this time, if nothing has been done to relieve the complaint, the patient cannot stand on the leg without pain; the tumour is now much enlarged, and conveys a distinct feeling of fluctuation; if an incision be now made into it, the surgeon will be surprised, and perhaps not a little mortified to find, that much *blood*, but no *matter* flows, and the wound *may* put on a healthy appearance, and heal slowly as a simple ulcer, but leaving behind a considerable cicatrix. This, however, is not the treatment I would recommend, having seen it produce an ulcer of very long standing.

The plan I pursue is to apply leeches to the tumour in the evening; when they fall off, a poultice is applied, into which the blood flows during the night. In the morning, the leg being washed, a compress of lint dipped in cold water or a solution of alum is laid on the tumour, over this a piece of oiled silk, and the entire leg from the foot to the knee carefully bandaged. In a couple of days, if the part becomes painful, the same treatment is to be adopted, and persevered in from time to time until the tumour subsides. At the same time I recommend cold bathing, if in summer, and a general tonic plan to be pursued, due attention being paid to the state of the bowels: during this time riding or carriage-exercise may be ordered, and even gentle walking when the tumour is not painful on standing. In this manner I have successfully treated this disease, (at a time that high surgical authority had deemed the *incision* of the tumours to be necessary) and with a comfort and liberty to the patient that could not be enjoyed, had the latter method been pursued. The tumours gradually lessen under this plan, and sometimes, not sooner than six weeks, is resolution effected, though even then a certain thickness remains in the skin. At this period a little camphorated mercurial ointment, rubbed into the part, will prove useful. The application of leeches frequently is troublesome, yet they are a slight inconvenience, compared to the pain of operation and long subsequent confinement. This disease arises in persons of a cachectic habit; as I have only seen it in those of the better ranks, and in females, I have at times been inclined to attribute its remote cause to wearing tight garters; I have not seen it described, but as I have no doubt that its immediate cause is the rupture of a small blood-vessel in the cellular substance under the skin, or a varicose vein producing subsequent inflammation, I would venture to apply to it the designation of *erythema hæmorrhagica.*

The following distinguishing traits, then, exist between the two diseases:

The erythema *nodosum* is ushered in by *febrile symptoms:* the appearance of the eruption is *sudden.* The *fronts* of the legs are the parts affected: the arms likewise may and do exhibit the disease, each blotch runs through its *inflammatory course,* if I may so call it, in two or three days, and depletion (I do not include bleeding) is indicated for its cure.

The erythema *hæmorrhagica* (as I have ventured to designate the other disease) is *not* preceded by febrile symptoms, it comes on *gradually;* it affects the *legs only* so far as I have seen, and the *calves* of these; none of the *characteristic* appearances of colour, as in the former disease, are exhibited; it may be weeks going through its course; the *tonic* plan is indicated for its removal; and it appears to be much more a *local* than a *constitutional* affection.

To conclude: the *former* disease seems to me to depend on an effusion of lymph;* the *latter* to a vein in a ruptured or varicose state causing topical inflammation."

There is yet another cutaneous affection, liable to be confounded with the erythema nodosum, yet differing from it in numerous respects. Mr. Blackley makes no mention of it, although it is extremely common. The disease is first noticed as a round, hard lump, like a marble, under the skin. The latter is at first quite undiscoloured, but, as the subcutaneous lump augments in size, it becomes adherent to the skin, and an erythematous blush is suffused upon the latter. If the tumor be incised in this stage, it is found to present a yellowish structure, like that of cheese, enveloped in a sort of cyst, formed by the cellular membrane. The tumor softens; the skin becomes redder, grows thin, and ulcerates; the structure of the tumor, half matter, and half a cheesy-looking unorganized deposit, is disclosed; this gradually sloughs out, and finally a cachectic ulcer is left. These subcutaneous tubercles are most common on the legs, though they do appear elsewhere. All do not advance to suppuration, some being spontaneously, or under appropriate treatment, resolved. They occur in persons much out of health, not unfrequently as a cachectic venereal secondary symptom. Some persons have named this the "subcutaneous tubercle." The best mode of treatment is the application of galbanum plasters and bandaging, as a local means; and mild purgatives, with tonics, especially sarsaparilla, and good diet, in the way of general treatment. When suppuration is evidently taking place, poultices, of course, are necessary, and usually it is better to allow ulceration to occur sponte suâ, in preference to employing the lancet or the knife. We have given this brief sketch of the "subcutaneous tubercle," in order to render the article more complete.

* "Some authors believe that *blood* is effused in erythema nodosum, and have therefore classed the disease with *purpura hæmorrhagica.* I do not think they are correct."

The affection is a curious, a common, and an important one, and justice cannot be done to it in a paragraph.

---

### THE CAUSE OF SLEEP.

Many of our readers are aware that a sharp controversy has existed, for some time past, between Messrs. Macnish and Carmichael, respecting the cause of sleep. The following extract embodies the chief points of Mr. Carmichael's doctrine.

"The absorbents and secerning vessels never remit their offices—those carrying off the old particles from every part of the frame, and these depositing new ones in their place; the absorbents being most busy with the muscular fibres which are most exercised by labour, or the nervous fibres most exercised by the operations of sensation, volition, and thought. Yet these fibres, so exercised, are always the strongest and most powerful of their kind in the frame: the secerning vessels must, therefore, be equally busy in restoring new particles in the place of the old, or during certain intervals, rather more busy, because more are restored than are taken away, as is proved by the increase of size, proportioned to the occasional or habitual exercise of the parts. Yet it is evident that it is not during the moments of exercise that the great mass of new matter is deposited, otherwise the muscular and nervous fibres in question would go on thickening and strengthening, the longer the exercise of labour and thought was continued; and this, we know, is contrary to fact;—fatigue ensues, and rest is necessary, and during that rest it is probable that the secerning vessels, though always depositing new particles, deposit much more, or the absorbents remove much less, than at other times. By rest, I mean a mere cessation from labour; and such rest is not sleep. The large mass of new particles deposited on the muscles cannot affect their tough and insensible fibres by any striking phenomenon; but when such a mass is deposited on the delicate, tender, and sensible structure of the brain and nerves, how different must be the effect. If small in quantity, and while these organs are in a state of active energy, it may be hurried unobserved into the existing activity of the living matter; but if large in quantity, and while these organs are resting from their labours, can it be that the extraneous and unassimilated mass does not press its increasing weight on their fragile machinery, and produce an EFFECT something *like* the pressure of the overswollen bloodvessels, but natural, necessary, and healthful—the PARALYSIS, not of apoplexy, but of SLEEP?

While the incumbent mass thus paralyses the *encephalon*, the body is powerless; there is no voluntary motion, no perception, no thought, no dream. But when the assimilation is complete in any one of the organs of the mind, then thoughts arise; but there is no perception until the assimilation is also complete in one or more of the organs of the senses; until then the simple current of our thoughts constitutes an ordinary *dream*.

If the nerves of motion continue invested in a newly deposited mass of nervous matter, while the mind anxiously desires and essays in vain to move the limbs—this is *nightmare*. If these nerves are extricated from their trammels, and those desires and efforts of the mind still continue—if they command and the nerves obey—this is *somnambulism*. But these dreams, whether ordinary and natural, or attended with the horrors of nightmare or the perils of somnambulism, vanish as our senses admit the impressions of the external world. We are then *awake;* but while thus awake, if the nerves of motion are still asleep—if their trammels still continue upon them—this is the *daymare*, so feelingly described by Mr. Macnish. If through any idiosyncrasy the process of assimilation were never sufficiently considerable to paralyze, by the mass of new particles, the brain and nerves of sense, the individual would exist as one that *never slept*, even though his nervous system should obtain, in some degree, those blessings which are the peculiar concomitants of sleep, a sufficiency of nourishment and a renovation of vigour. If, through an opposite idiosyncrasy, the deposit of new particles should be so superabundant and incessant as to continue the paralysis beyond the usual and natural period of slumber, this state would present the rare and hitherto mysterious phenomena of *protracted sleep*, sometimes terminating even in death, as in the case of Elizabeth Perkins, detailed by Mr. Macnish. These two opposite idiosyncrasies seem to arise from opposite diseases of the secerning vessels of the head, one promoting to excess, and the other in an equal degree preventing, the effusion of the due quantity of nervous matter requisite for the healthy and vigorous state of the nervous system.

If it should be asked, How can the same cause operate in different ways? How can the assimilating process at one time cause sleep, and another not cause it? How can it, though unremitting in activity, at one time paralyze the brain and nerves, and at another rather enliven and invigorate them?—These questions are difficult, and the more difficult because, in the material world, we can find no object wherewith to compare and illustrate the phenomena of mind. The element of fire must suffice on the present occasion, where no better ligament of analogy between things so different can be had:

'Nutritur ventis, ventis extinguitur ignis;

Levis alit flammas, grandior aura necat.'

If a fire burns clearly, brightly, and fiercely, still it requires a constant supply of fuel to keep up its intensity, and replace the solid particles expended in combustion. A small quantity frequently added, so far from paralyzing, increases the activity of the fire; but when that activity is exhausted, when the very energy of the flames, like the exertion of a powerful mind, has wasted away the substance on which it fed, and these flames sink enfeebled, and the fire is diminished and dull, if you heap over it a heavy mass of fuel, the flames are smothered, the activity ceases, the element sleeps. Hours are required to extend the vivifying influence to the new matter; at length the increasing warmth pervades the whole mass, the assimilation is complete, and the smallest incitement stirs up again all the energies of the furnace. If too little aliment be supplied to the glowing mass, it will burn out, like an overworked brain in similar circumstances; while too great a weight of fuel cast on the exhausted hearth overwhelms the expiring embers, and the result is the slumber of death, not of sleep."*

The hypothesis (for we believe it may be considered only as hypothesis) of Mr. Carmichael is ingenious—perhaps it may be true. The laws of alternate sleep and activity, like all the laws imposed on us by the Creator, are, no doubt, the wisest and the best that could be devised. We can trace the operations of many of them—but very few of them can we explain. It strikes us that there are some difficulties in the way of Mr. Carmichael's explanation of the causes of sleep. If this image of death be occasioned by the deposition of large masses of new matter on the delicate organization of the brain and nerves, inducing the "paralysis of sleep," it is curious how the bite of a flea or a bug will often disperse all these depositions, and start us into wakefulness! It is also curious that all animals become more or less sleepy, during the very first process of *digestion*, in the stomach, and long before assimilation, much less deposition of assimilated matters, can possibly commence. The dog, the hog, and the glutton fall fast asleep as soon as the stomach is crammed to satiety. They are most awake and active after digestion, and when assimilation, secretion, &c. are in full play.

Then, again, extreme cold and hunger induce an almost irresistible propensity to sleep. Does this arise from depositions of matters on the brain and nerves, for the renewal of their parts and powers?

It appears to us that every thing is periodical or alternate in this world,

* The Phrenological Journal, June 1, 1835.

and nothing constant in action, even for a short time. The muscles cannot always be in a state of contraction. The brain cannot always be thinking. But the organ of the mind cannot cease to think, except through the mysterious agency of sleep—and, therefore, sleep was ordained. How it is induced we know not. Opium will often lull to repose by diminishing sensibility; but still we are totally ignorant how the sleep is produced in consequence. We must, therefore—

"Wait the great teacher Death, and God adore."

---

### Dr. Mac Adam's Case of Laryngitis.

Dr. Mac Adam relates this case in our Dublin contemporary for July, because he thinks it offers evidence of the value of the operation of tracheotomy, and because the post-mortem appearances seem to him to throw light on the immediate cause of death in laryngitis, when it occurs sometime subsequently to the operation.

*Case.* Christopher Nowlan was admitted into the City of Dublin Hospital, on the 16th of March, 1835, with the ordinary symptoms of laryngitis. By the stethoscope, placed on the larynx, a loud, harsh, crowing sound is heard, extremely grating to the ear; the chest sounds natural on percussion, both anteriorly and posteriorly, and the loud laryngeal sounds, re-echoing through the thorax, prevent any respiratory murmur or other sounds from being distinguishable; there is some sonorous râle, however, audible in the posterior part of the chest, though nearly drowned by the laryngeal sounds. He complains of soreness when pressure is made on the alæ of the thyroid cartilage, which also excites a fit of coughing. There is no aphonia, his voice, though extremely hoarse, can be heard at some distance distinctly. Pulse 108, soft and compressible; tongue clean; there is some slight redness of the fauces, without tumefaction; face slightly flushed, bowels confined.

Seven weeks previously, he had been attacked with fever, which lasted for three weeks. On the decline of the fever, he became affected with cough and sore-throat. These symptoms had become aggravated into the intensity described on the night of the 15th.

They continued, with little change, from the 16th till the night of the 17th, when they became much aggravated. At half-past twelve o'clock in the morning of the 18th, Dr. Mac Adam was sent for, and found the patient much worse; he appeared in momentary danger of suffocation. The dyspnœa increased, and, at half-past 1, a.m. he seemed sinking. Tracheotomy was now determined on, and peformed, the patient seeming actually dying at the time. The upper rings of the trachea were rapidly laid bare—the wound, which was deep, was filled with venous blood, which continually welled up from its bottom. Dr. Benson, the operator, fearing to use the knife under such circumstances, plunged a large trocar into the trachea, expecting that the canula would so fill its transit as to prevent any blood from getting in; this had the desired effect, as was evident by the firmness with which the instrument was grasped by the elastic trachea.

"On the introduction of the canula into the trachea, a strong inspiration ensued, followed by a violent fit of coughing and dyspnœa, succeeded by the expulsion of a quantity of viscid mucus mixed with blood through the tube; almost immediately after which his respiration became comparatively easy, the colour returned to his cheeks, his pulse became more distinct, and his surface warm, and in a short time he was able to speak distinctly, to sit up in his bed, and to take some drink. He had, however, frequent attacks of violent cough and dyspnœa, succeeded by the expulsion of bloody mucus through the tube, followed by nearly natural respiration, which was evidently performed exclusively through the canula, for a lighted candle brought near its orifice was extinguished during expiration; and when the finger was placed on its extremity, respiration was suspended, and the patient suffered the distressing sensation consequent upon holding the breath."

During the day and night of the 18th, there was little change. Great care was necessary to keep the tube free from the viscid mucus, with which it was incessantly obstructed. On the morning of the 19th, he was salivated by mercury, which he had taken for forty-eight hours. The canula slipped out, and it was necessary to re-insert it. Debility increased, although no great change occurred in the other symptoms. Early in the morning of the 20th, about 53½ hours after the performance of the operation, he expired.

*Dissection.* "The muscles being carefully dissected off the larynx anteriorly, the sterno-hyoid and sterno-thyroid were found matted together by coagulable lymph, and could scarcely be distinguished from each other. The tongue, trachea, and lungs were removed together from the body, for the purpose of a more minute examination. The opening made by the trocar into the trachea was distinct and gaping, and was found situated between the first and second rings, penetrating the lower part of the thyroid gland. On raising this gland a considerable mass of semi-cartilaginous substance was found covering both alæ of the cricoid cartilage; on dividing this substance, an abscess was exposed lying on both sides of the cricoid, which was denuded anteriorly; both alæ appeared sound; this cartilaginous mass occupied the situation of the crico-thyroid muscle. On dividing the pharynx posteriorly, the whole anterior surface of the epiglottis was found elevated by serous effusion into the submucous tissue, appearing like a gelatinous mass between it and the root of the tongue; the effusion extended along the aryteno-epiglottidean folds; the arytenoid cartilages were covered with the same substance, and appeared greatly enlarged and altered in form. On looking down into the larynx, the rima appeared completely closed; the ventricles were obliterated by the elevation and thickening of the mucous membrane above and below; the chordæ vocales were thickened and rounded, not presenting a defined edge. The back of the pharynx and trachea being divided between the arytenoid and broadest part of the cricoid, the abscess, which was before seen on the sides of the cricoid anteriorly, was now found to extend all round the posterior part of that cartilage; the same semi-cartilaginous structure was observed in the sac of the abscess posteriorly as anteriorly. This portion of the abscess occupied the place of the posterior crico-arytenoid muscle, its cavity was of a dark greenish colour, the size of a bean, and exhaled a gangrenous odour. There was no communication between this abscess and the cavity of the larynx, but it opened into the pharynx, about one inch and a quarter from the tip of the arytenoid cartilage. The cricoid cartilage was partly ossified and extensively carious; all the interior of the trachea was lined throughout by a gelatinous effusion of a reddish colour, appearing in some parts like coagulable lymph, and every where very adhesive; on scraping it away the tracheal mucous membrane was found softened and highly vascular, presenting a pale appearance in the larynx, but was of a light pink colour at the root of the epiglottis. There was no ulceration detected on any part of the internal surface of the larynx or trachea."

The same kind of gelatinous fluid noticed in the trachea, was also found in the right and left bronchus, the ramifications of the former being filled, even to those of the third and fourth order. In the upper part of the right lung were a few detached tubercles, some softened, and the bronchial glands were indurated and enlarged.

The disease in this case had probably, we might almost say had evidently, been chronic disorganization of the cricoid cartilage, terminating in abscess, and caries of that cartilage. On this supervened fever, and, on the fever, the acute laryngeal inflammation. Dr. Mac Adam is of opinion that the operation was decidedly of service. A consideration of the post-mortem appearances explains, he thinks, the immediate cause of the patient's death. The right bronchus was completely plugged up, and the left nearly so, with a gelatinous effusion, which also lined all the interior of the trachea, proving, that intense bronchitis had existed, and rendering it evident, that the immediate cause of death was obstruction of the bronchial tubes, the consequence of effusion produced by inflammation of their lining membrane. While the patient retained sufficient strength to perform the respiratory efforts necessary to overcome this obstruction, and to expel a portion of the viscid mucus, enough of air was inspired to preserve life; this, however, was progressively diminishing, according as the effusion increased, and the patient's strength declined. The circulation also became gradually depraved, in consequence of the less perfect oxydation of the blood; another debilitating cause arising from this source was added to the exhaustion consequent upon long-continued laborious respiration, and the patient sunk when his powers ceased to be equal to those combined debilitating influences. Bronchitis, then, and its consequences, were the immediate cause of death, nay Dr. Mac Adam seems disposed to think, that, had the bronchial inflammation been detected the day subsequently to the operation, local depletion, blisters, antimonial solution, and so forth, might have even protracted the duration of the patient's life. In this we fear the Doctor is too sanguine. "If I should meet with another case similar to the one I have been just considering, I would be disposed, very soon after the operation, to apply leeches to the upper part of the sternum, followed by a blister, and to put in practice the general treatment suitable to acute bronchitis, as far as circumstances permitted. In this case, the patient would probably have ultimately died, even if the bronchitis had been subdued." In the latter opinion, we agree with the Doctor. The disease in the neighbourhood of the larynx had proceeded too far to permit much rational expectation of success from any mode of treatment. The case is interesting pathologically as well as in a surgical point of view.

---

### OCCLUSION OF THE VAGINA.

The following is a curious and not uninteresting case, because it exhibits the possibility of conception although the vagina was closed to a very considerable degree. It is contained in our esteemed contemporary, the American Journal of the Medical Sciences, for February, 1835. We must give it in the words of the contributor, Dr. Hoillemin of Aux Cayes, Hayti.

*Case.* "Madame —— de ——, when twenty-five years of age, had an exceedingly difficult labour, lasting three days; during which she had no other assistance than that of an inexperienced midwife. The external parts of generation, as well as the vagina, were attacked with violent inflammation, which was followed by an almost complete closure of the vagina, only a small opening remaining, scarcely sufficient to allow of the passage of a goose quill. She long suffered from incontinence of urine, and much difficulty in walking.

About June, 1830, Mad. ——, then twenty-seven years of age, first consulted me. She was at that time suffering with nausea, loss of appetite, progressive increase of the abdomen, swelling of the breasts, &c. I immediately recognized all the symptoms of pregnancy at the third or fourth month, and informed Mad. —— of it, who replied that it was impossible for her to be pregnant, since she could not cohabit with her husband, because her parts were closed, "*ses parties sons fermé*," (this was her expression.) The husband, who was present, confirmed all that his wife had said. Nevertheless, I assured her, that she was undoubtedly pregnant, and I did my best to tranquillize her great uneasiness, for she incessantly repeated that it was impossible for her to give birth to her infant.

The 30th of December of the same year, Mad. —— sent for me at midnight. Labour pains had just come on. On examination I found that the vagina was closed by a firm membrane, extending across it, and which was thickest laterally. Near the meatus urinarius, a kind of fleshy band originated, which was lost in the partition. In the centre of this last there was a round opening, scarcely large enough to admit a quill, and the margin of which was thick.

I proposed to Mad. —— to divide the membrane closing the vagina, to which she consented. After the uterine contractions had continued for six hours, I took advantage of the moment when the membrane was pressed forward and downwards by the membranes, and the head of the child to divide the margin of the opening, and then inserting the index finger of my left hand between the head of the infant and the partition, with my right hand, I passed the blade of a straight probe pointed bistoury upon the finger which served as a conductor, and cut the membrane from within outwards, on the left side, to the extent of an inch, and then waited the effect of the renewal of the uterine contractions. After an hour, during which these were strong and frequent, the opening not enlarging, and the membranous partition being constantly pressed down, I made another incision from within outwards on the right side, so that these two incisions formed a triangular flap, the base of which was towards the sacrum. The umbilical cord immediately protruded; the waters, which were discharged, were black, and exhaled a strong and disagreeable odour. Mad. —— became covered with a cold sweat; had repeated faintings; her pulse was almost imperceptible, and the uterine contractions were infrequent. Suspecting that the child was dead, from there being no pulsation in the umbilical cord, and having great fears for the mother, I hastened to terminate the labour by delivering with the forceps. The child appeared lifeless; its surface was livid, indicating cerebral congestion. After dividing the cord, I allowed three or four ounces of blood to flow; I employed dry frictions over the cardiac region, &c. and was not a little surprised to see the infant revive, as well as the mother, both of whom are at present in the enjoyment of perfect health.

Precautions were taken to preserve separate the parts which had been divided, and to prevent their reunion; the opening of the vagina was thus re-established in its natural state. The triangular flap resulting from the two incisions gradually diminished, and at the end of two years no trace of it remained, and Mad. —— could cohabit with her husband without experiencing any inconvenience."

The case is deserving of attention.

---

### Melancholy Effects of Inordinate Depletion.

Professor Badham has recently related the case of a lady who appears to have been most profusely bled for *severe pain* in the head—and which, in all probability, was not accompanied by much, if any inflammation. The lady was 30 years of age, and five or six months advanced in pregnancy. She had experienced preceding abortions, and nearly lost her life by uterine hæmorrhage. For many years, too, she had suffered from severe hereditary headaches. By these affections, and the remedies employed, she was reduced to a state of great debility, her lips being blanched, and her countenance indicative of distress. She was attacked by one of these constitutional head-aches, of a violent kind, and medical assistance was called in. The preliminary reflections of Dr. Badham, on the mistaken opinion which the medical man formed, on this occasion, we shall omit, considering them much too severe.

" One large bleeding from the arm (the blood from which exhibited no buffy coat) was succeeded by one hundred leeches at various times, for auxiliaries; this was the practice. The blood estimated to have been taken (during five days) was at first put at seventy ounces, but reduced to sixty (almost four pounds avoirdupois) by a later computation—an estimate which, however, I dare venture to consider as much below the mark, when I look at the agents employed. As to the leeches, blood thus drawn was not, it seems, to be scrupulously considered, or taken at its apparent value. Alas! how much fatal mischief has this current absurdity of medical men not produced? and when will the unmeasured drain occasioned by leeches be looked upon as it ought, with more suspicion than venesection itself? That not the slightest relief was obtained may sufficiently prove the error of the application here; that the whole of the distressing consequences now to be stated, should have ensued, will occasion surprise. Had abortion been the only one—had it not been, in fact, the very slightest of those consequences—this admonitory narrative would have had no existence. Those consequences were, I admit, unusual; but I believe many of them to be producible just as certainly, and as often, as such a practice shall be adopted on such a subject. Spasms, all but tetanic, of internal as well as external muscles, and attended with excruciating pain, placed her existence, for full a fortnight, in hourly danger. I found the unfortunate sufferer, at the end of even four weeks, still clenched and screwed by these spasms (now, however, become very different, from the frightful violence of the still earlier days of her misery) to an extent most distressing to witness. I found her drenched in incessant sweat, and in such a state of deplorable debility, that she could not move her own limbs in bed, nor raise her head ever so little, if it rolled from the pillow; added to all which, that almost entire privation of sleep, which had allowed no respite to her pains for fourteen days, still continued to resist the various narcotics of which trial had been made.

The abortion of a six month's child had taken place on the 14th or 15th day from the attack, and on the 8th or 9th after the last of the bleedings; and it is worth while to remark, that the system was, at this time, so drained of blood, that, the expulsion accomplished, not enough blood followed, either then or subsequently, to stain a handkerchief. But far sadder consequences than all these are still to be related. Two or three days after the abortion her vision appeared to be failing, and this fatal suspicion was soon confirmed. The last object in the material world that she ever saw, was the bright yellow gown of her female attendant.

At that period, I should have mentioned, when the spasms, perfectly uncontrolled by common remedies, were plainly destroying her, and when it was obviously impossible that life could resist much longer, an introduction of the most powerful medicines was commenced, under the personal superintendence of a medical friend, who came from London to her assistance, and, as I believe, saved her life by musk, opium, belladonna, &c.

But the measure of her calamities was not yet full; the senses of smell and of taste were also found to be perishing; and perish they did,—the first entirely and permanently; the second has to a certain extent recovered. Touch, together with all prehensile power, followed, or departed, with the others, and of course (for touch is the sight of the blind) greatly aggravated her misery. She did not know when her hand was pressed by another's, or her pulse felt. Her arms and fingers were in perpetual cramp; and for one night she complained so much of difficulty of swallowing, that I was seriously apprehensive of tetanus; the rather as she had pains in the lower part of the spine, of great severity, and only capable of relief by the most violent pressure we could apply. As to the original pain of the head, it had left her; but it was succeeded by noises which never ceased, 'like the din of a great manufactory, or the noise of a thousand anvils,' together with what might seem an incompatible sensation of emptiness, 'as if the brain ached for want of blood*.' From the beginning her intellect had never been disturbed for one moment; neither disturbed nor excited; it was calm, collected, and equal to her situation; most sensible to the attentions paid to her; desirous of sparing the fatigue of those around; affectionate to the objects of her affection; considerate for all. But the headache did not remain without some probable explanation.

* See Dr. Bright on this subject.

Long after the mischief was done by blood-letting, many quarts of dark-coloured caustic bile, which scalded as it passed, appear to refer the source of her original sufferings to disordered or suspended secretion of the liver.

Her life was spared. A slow convalescence, with many checks, at length began to take place; but though all the other functions have returned, the organs of sense have never rallied. She is utterly, and, at the end of six months, hopelessly blind; she cannot yet discern by touch any difference of surfaces, nor the difference of size between a large and a small coin; she cannot perceive any odours whatever of flowers or aromatics; she cannot smell a cigar, but can just smell ammonia."*

The above is a very melancholy case. We doubt, though we do not deny, the inferences which Dr. Badham has drawn. We doubt these inferences, because, in a long professional life, where the sphere of observation, public and private, was at least equal to that of the author, we never met with an instance where the loss of four pounds of blood, under any circumstances, occasioned such remarkable phenomena as are recorded in the foregoing case. On the other hand, we have seen several cases, in females, where a train of symptoms, not very different from those of this unhappy lady, occurred, without the loss of a single ounce of blood. We have seen the lower extremities paralyzed for years, accompanied by the most singular aberrations of different senses—all from hysteria—and all vanishing in the course of time. We hope and believe that the unfortunate case narrated by Dr. Badham will turn out to be one of these. But, at the same time, we dare not deny the justness of Dr. B.'s conclusions. It is possible that, in this case, the abstraction of so much blood may have led to the deplorable consequences which have been somewhat poetically portrayed by the narrator; but we think that he has not exhibited an exuberance of charity for the frailties of human nature, and for the errors of judgment to which we are all liable. If it was capable of demonstration that the practitioner had completely mistaken the above case, Dr. B.'s language would still be too strong; but when we reflect that the error of judgement is NOT proved—what shall we say to the harsh JUDGMENT, which a brother practitioner has pronounced against his neighbour? No one will accuse us of the attempt to excuse delinquency, ignorance, or temerity; but for mere *errors of judgment* we ought to have some compassion. The PUBLIC are quite prone enough to condemn us, on their own shallow reasoning—we ought not to put weapons in their hands, loaded with extraordinary charges, that are not perhaps legal or honorable after all.

* Med. Gazette, July 25th, 1835.

---

### On the Employment of the Stethoscope in the Diagnosis of Aneurisms of the Thoracic Aorta. By G. Greene, M.D.*

The diagnosis of aneurism of the thoracic aorta is confessedly obscure, and the majority of practitioners have both seen and committed mistakes in relation to it. Those acquainted with auscultation are aware that Laennec estimated the powers of the stethoscope in the investigation of diseases of the heart and arteries, at too low a rate, and probably he ranked its merits in the discrimination of diseases of the lungs too high. The object of Dr. Greene in the present paper is to shew that auscultation does really afford very valuable aid in enabling us to distinguish aneurism of the aorta. He relates three cases.

Case 1. *Double Aneurism of the Thoracic Aorta—Sudden Death from Rupture of the Sac into the Left Bronchial Tube.*

" A sawyer was admitted into the Whitworth Hospital, House of Industry, on the 7th of April, 1834. He had been subject for the last two years

* Dublin Journal, May 1835.

to a cough, attended with great dyspnœa, and frothy expectoration. The cough seized him in paroxysms, generally at night. He also complained of irregular pains, of a lancinating character, through the chest. These symptoms became so severe and unremitting, that he was obliged to discontinue his business six months previous to his admission."

When Dr. Greene first saw this patient, the cough would last for half an hour at a time, and threaten suffocation. The slightest exercise brought on dyspnœa—there were darting pains in the chest, though the greatest distress was referred to the summit of the sternum—the breathing was laryngeal—there was slight dysphagia—turgescence of the left jugular vein—no external tumor nor visible pulsation.—Such was the history, such the general symptoms. Let us now regard the ausculatic indications.

"The chest sounded clear all over, on percussion; on applying the stethoscope, a distinct impulse was communicated to the instrument over the upper third, and to a short distance on either side of the sternum. The impulse was much stronger than that of the heart; it was not attended with any distinct bruit; respiration in left lung feeble, loud and clear in the right, especially on a forcible inspiration. There was also a distinct impulse communicated to the instrument for a considerable distance down the back, to the left of the spinous process of the vertebræ; no purring tremor could be detected; a distant but distinct bruit de soufflet in this situation; impulse and sounds of the heart normal. When the instrument was applied to the axilla, or to the back, in the situation mentioned, the respiration appeared to be almost laryngeal in the top of the left lung, but was natural in the right, and loud and distinct."

On the morning of the 12th of April, the patient was suddenly seized with hæmoptysis, accompanied with intense pain in the mammary region of the left side. A sort of mucous râle was audible in the top and centre of the left lung. In half an hour the patient died, with the lips and extremities cold and exsanguined.

*Dissection.* The heart was sound. "On cutting into the substance of the left lung, its parenchymatous structure was found to be injected with blood, which was also found in great quantity in the pleural cavity. The right lung was similarly injected in its posterior half, its anterior edge was emphysematous. The injected portions could easily be broken down by the finger. The aorta was enlarged, and on slitting it up atheromatous deposites were found between its inner and middle coat. From the anterior surface of the vessel, at the point where it winds round the left bronchial tube, an aneurismal sac was discovered about the size of a small orange; it communicated with the left bronchial tube by an opening just large enough to admit a probe. The interior was lined with fibrinous deposites, and it had slightly compressed the left bronchial tube. The œsophagus was situated to the right of the tumour. On removing the lungs, another aneurism on the anterior surface of the aorta was discovered, somewhat larger than the first, and corresponding to about the seventh dorsal vertebra. It had contracted strong adhesions to the posterior surface of the left lung, and the interior was also lined with fibrous deposites similar to the first."

CASE 2. *Aneurism of the descending portion of the Aorta. Sudden Death without Rupture of the Sac.*

Ann Lee, æt. 27, admitted into the Whitworth Hospital, Jan. 8th, 1835. About two months previous to her admission, she had fallen down a flight of stairs upon her back, since which she had suffered pain in that situation. About six weeks before admission, she became affected with cough and dyspnœa, succeeded by difficulty of swallowing.

When Dr. Greene saw her, the cough was loud, harsh, of a peculiarly ringing character, and attended with a frothy and rather copious expectoration. It seized her in paroxysms, during which she was almost suffocated; it was particularly severe at night. There was dyspnœa—difficulty in swallowing, referred to the upper third of the sternum, where the morsel seemed to stop, and required the aid of liquid to descend into the stomach—in inspiration the trachea was drawn deeply down behind the sternum—pain on pressure of the spinous processes of the third and fourth dorsal vertebræ, and pain, also, shooting from the sternum to the spine. Pulse 98, full, and regular—respiration hurried—catamenia deficient for the last six months.

"The following were the signs furnished by the stethoscope: A clear sound obtained all over the chest on percussion; the respiration in the right lung distinct, but accompanied by a kind of blowing sound, when the instrument was applied posteriorly, about the situation of the bronchial tube, or in the axilla of the right side. The respiration in the left lung was feeble, and sometimes nearly inaudible. An obscure impulse, accompanied by a bruit de soufflet, in the interscapular region. Both these phenomena most distinct at the left side of the spine, and confined to the situation where she complained of pain on pressure: they were most remarkable when the arterial system was excited by any sudden exertion. Anteriorly an impulse was communicated to the stethoscope at the sterno-clavicular articulation, and sub-clavian region of left side. The impulse was attended with a double sound, and was somewhat stronger than that of the heart. An obscure bruit de soufflet was heard in the same situation; the impulse and sounds of the heart were natural. She complained of pain when percussing the top of the sternum."

We need not particularly mention the treatment, which was antiphlogistic. Two days before her death, which occurred suddenly in the night of the 5th February, another stethoscopic examination was made, and as some alterations in the phenomena had occurred, we subjoin the results of the exploration.

"A strong double impulse a little below the sterno-clavicular articulation, at the left side of the median line of the sternum, accompanied by two distinct sounds, and a clear bruit de soufflet. Impulse diminishes towards the heart, which retains its natural impulse and sounds. No expansive pulsation found on pressing the fingers deep behind the clavicles; left jugular vein turgid. Posteriorly, at the situation of the third and fourth dorsal vertebræ, a decided, and, apparently, a single impulse, accompanied with a bruit de râpe, communicated to the instrument. These signs diminish in intensity as the stethoscope is applied down the spine at the left side. Respiration presents the same characters as on the first examination, but more clearly marked. On pressing the ball of the thumb forcibly to the spine, at the situation of the impulse, a tremor is communicated to the hand; the pressure produces pain. On looking along the anterior surface of the chest horizontally, a distinct elevation perceptible at the top of the sternum, where percussion also produces pain, and a pulsation can be felt; no dulness in the situation of the impulse."

*Dissection.* The heart was sound. An aneurismal tumor was discovered arising from the descending portion of the arch of the aorta, where it is in contact with the left bronchial tube, and stretching across the vertebræ to the right side. The œsophagus ran directly in front of the tumor, separating it from the bifurcation of the trachea. At the junction of the transverse with the descending portion of the aorta, a smaller aneurism, large enough to contain a bean, extended behind, and pressed upon the left bronchial tube, between it and the œsophagus, and communicated with the aorta by a well defined opening. The larger tumor rested upon the bodies of the third and fourth dorsal vertebræ; the osseous portion of which was absorbed, leaving the intervertebral substance prominent. This portion of the spine formed the posterior wall of the sac, the pressure of which had somewhat flattened the right bronchial tube. The sac communicated with the aorta by a well-defined opening, large enough to admit a man's thumb. Its interior was lined with a firm fleshy-looking coagulum, and did not appear to contain much recently coagulated blood. Its size was about that of a small orange, and the walls were perfect.

CASE 3. *Aneurism of the Thoracic Aorta—Sudden Death from Rupture of the Sac into the Left Bronchial Tube.*

This case is possessed of the more interest, as an accurate diagnosis was made at an early period by means of the physical signs.

Michael Hughes, æt. 38, was admitted into the Meath Hospital under the care of Dr. Stokes. Five months previously he caught cold, and shortly afterwards became affected with pains in the back and sides, and with dyspnœa. These symptoms increased. On admission the dyspnœa was great—there was cough, loud and ringing, and of a peculiar croupy character—scanty expectoration of frothy mucus—pulse 86 and full. The auscultic indications were as follow:—

The whole of the chest sounds clear on percussion. Respiration in the right lung intensely puerile, altogether absent in the superior portion of the left, and only feebly audible in the inferior portion. On applying the stethoscope to the left axillary region, and the patient being desired to draw in a deep breath, no sound is heard during the first half of inspiration, but during the latter half the air appears to overcome some obstruction to its entrance, and suddenly rushes in to expand the lungs. The left side of the chest is almost immovable during respiration, but the right acts freely. On applying the hand to the upper part of the left side, no vibration of the voice could be detected. The vibration is very feeble below on the same side, but well marked on the right side; impulse and sounds of the heart natural; a strong double pulsation is heard over the sub-clavicular region of the left side, also over the postero-superior portion of the same side, and on applying the hand over the former situation a distinct impulse is communicated to it; no bruit de soufflet could be detected.

A short time after this, a strong double pulsation could be heard across the right clavicle, and the pulsation in the sub-clavicular region, as well as over the scapula had increased, with a strong impulse below the middle third of the clavicle; after the patient had walked several times up and down the ward, a sharp bruit-de-scie was detected in the latter situation. When the patient remained quiet for a short time this disappeared, but again returned after exercise.

Subsequently the patient was admitted into the Richmond Hospital, and again an examination of the physical signs was resorted to. These are the particulars.

Anteriorly the right side of the thorax sounds well on percussion; a little dulness in the sub-clavicular and mammary region of the left side; respiration in the right lung remarkably loud and clear, very obscure in the left lung, except on taking a deep inspiration, which produces a feeble vesicular murmur. A strong impulse communicated to the stethoscope, two inches below the clavicle, and about an inch and a half to the left of the median line of the sternum; impulse also perceptible across the sternum to the right clavicle; it is greater than that of the heart, and appears to be double; it is also perceptible to the hand. A slight impulse communicated to the stethoscope at the left side of the second and third dorsal vertebræ; no bruit de soufflet could be detected in this situation, nor anteriorly except after great exertion; pain on percussion of the upper third of the sternum, and in the situation of the vertebræ above-mentioned.

We need not specify the general symptoms. They were as before, with the exception of being aggravated in degree. We shall state, however, that the impulse and sounds of the heart were natural. He expired suddenly in a fit of coughing, during which he spat up a quantity of blood.

On dissection an aneurismal tumor was found arising from the descending portion of the aorta, and pressing upon the left bronchial tube, which was considerably indented and narrowed. The posterior wall of the sac was made up by the bodies of the second and third dorsal vertebræ, the osseous portion of which was absorbed, and presented nearly the same appearances as in the case last detailed. The interior of the sac was lined with a fibrous coagulum, and it opened into the left bronchial tube; the œsophagus was slightly pushed to the left side; the interior of the aorta was crowded with atheromatous depositions between the internal and middle coat; the heart and pericardium were natural.

We have abbreviated or omitted all particulars not directly connected with the diagnostic evidence afforded by auscultation and percussion. The gist of the paper is this, and this only, that aneurisms of the aorta in the thorax may be distinguished with more facility by the aid of an examination of the physical signs than is usually imagined. This is the point which our author and ourselves are both anxious to insist upon, and irrelevant matters may be advantageously dispensed with.

We feel in doubt whether it is worth while to offer any remarks on an objection which has frequently been made to investigations and to cases of this nature, an objection as often refuted as advanced, and as often repeated as refuted. The enemies, for there are such, of pathology, always harp on the cui bono of the diagnosis of incurable diseases. What matters it, they say, if we do know that there is aneurism of a certain part of the thoracic aorta? Can the auscultators cure this better than their neighbours? The gentlemen who indulge in this philosophical train of reflection should recollect two circumstances not altogether unimportant. In the first place, if we determine that a malady is incurable, we are enabled, at all events, to avoid unnecessary or injurious interference, and to offer the patient or the patient's friends that accurate information which is often of benefit to them, and always creditable to the physician and his science. In the second place, it follows, as a logical sequitur and a practical result, that if we are able to determine diseases that cannot be cured, we are also able to distinguish those other diseases which can. Surely this is no small matter. We almost feel ashamed of arguing the question—we are tempted to indulge in an incredulous smile at the thought that any reasonable person can possibly object to whatever gives additional certainty to physic.

We may now revert to the subject more especially before us—the deductions which may be fairly drawn from the preceding cases. Those deductions are important to the physician who is anxious to obtain the greatest possible certainty of diagnosis. Let us hear what Dr. Greene has to observe.

"On examining these cases, it will be perceived that there were some symptoms common to them all. The first I shall consider is the remarkable difference in the respiration of the two lungs, and I am the more anxious to call attention to this fact, from its having been much insisted on as an additional means of assisting our diagnosis in this disease, in a valuable paper on the pathology and diagnosis of aneurism, by Dr. William Stokes, in the fifth volume of the Dublin Medical Journal.

The value of the sign, according to Dr. Stokes, consists in this, that the absence of respiration cannot be accounted for by any lesion of the lung, discoverable by auscultation or percussion. The fact of there being a clear sound on percussion, and at the same time a feebleness of the respiration, naturally leading to the conclusion that some impediment must exist to the entrance of the air into the lung on inspiration. He also suggests that perhaps it may be found, from further observations, that a solid tumour pressing on the bronchial tube will produce a permanent feebleness of respiration, but that an aneurismal tumour may allow of the occasional re-appearance of it, according to the variable nature of the contents of the sac. I could not perceive, however, in any of the cases above mentioned, that this latter phenomenon took place, but I found the fact of a feeble respiratory murmur, combined with a forcible entrance of the air into the bronchial tube on deep inspiration, of great assistance in forming a diagnosis."

Dr. Greene has seen one case of a solid tumor compressing the trachea. Yet the case was hardly of that description neither, for the tumor, an enlarged and diseased bronchial gland, was situated at the bifurcation of the trachea, on which it exerted no mechanical pressure. In this case there was no appreciable difference in the respiration of the two lungs. The patient had the symptoms of acute bronchitis.

The modification of respiration in a lung, in consequence of the pressure exerted by an aneurism on a bronchial tube or on the lung, is not adduced by Dr. Stokes as a pathognomonic sign of the disease. Any tumor capable of exercising mechanical pressure may produce the same effect. But if the general or other physical signs of aneurism be displayed, this assists very greatly in enabling us to form a more accurate opinion of the nature of the malady. In the inferior part of the descending aorta within the thorax, the pressure of an aneurismal or of any other tumor can scarcely be felt by a bronchial tube. The lung itself may be compressed, but the bronchial tube is too distant to be implicated.

" The next sign I shall consider as having occurred in these cases, is the fact of an anormal pulsation in the interior of the chest. It will be recollected that Laennec did not place much reliance on this symptom, except when it occurred under the form of a strong single impulse. Several circumstances, however, may prevent us from perceiving this single impulse, when the chest is examined anteriorly. The proximity of the tumour, for instance, to the heart, as at the origin of the aorta, will necessarily render the occurrence of this phenomenon less available for diagnosis, from the contiguity of the impulse of that organ. Again, should the tumour be situated at the descending portion of the aorta, its impulse will be masked by that of the heart which lies before it. These difficulties will be increased in hypertrophy of the organ; notwithstanding these obscurities, however, the sign alluded to is a most important one. It has been remarked by Dr. Hope, that when this phenomenon occurs posteriorly, particularly when accompanied with a sound of a rasping character, it is almost a positive indication of the development of aneurism, and I found this phenomenon to be well marked, particularly in the case of Lee."

Dr. Greene observes that two sounds may be heard in an aneurismal tumor. Of this there cannot be a question. In aneurisms of the ascending part of the thoracic arch, we have heard the two sounds with as much distinctness as in the heart itself.

" With reference to those symptoms which, in the language of systematic writers, are called 'general,' many differences must of course occur, according to the situation which the sac occupies. It will be observed, for instance, that in those cases no assistance was derived from a difference in the radial pulsations, or from numbness or œdema of the upper extremities. The absence of these symptoms, whenever the physical signs of an aneurism are strongly marked, may, perhaps, lead us with more certainty to a knowledge of the part of the vessel which the disease occupies, and also to the progress it is making. This is so obvious from considering the anatomy of the aorta, that I need not here make any lengthened observations upon it. In fact, when we have the physical signs above adverted to, and those general symptoms absent, we must infer that the tumour is below the origin of the great vessels going to the head and upper extremities; and if, in the course of the disease they make their appearance, we may infer that the sac is expanding upwards, and exercising a pressure on them. The same observation will apply to the absence of numbness and œdema of the upper extremities."

There cannot be a stronger argument in favour of the value of the physical signs, than the indeterminate and fallacious character of the general symptoms. Functional and organic affections of the heart, affections of the lungs, and aneurisms of the thoracic aorta, all produce symptoms of a similar or nearly similar character, though the functional or organic diseases that give rise to them vary very widely in character, in nature, and in severity.

Dyspnœa is one of those general symptoms. It was present in the cases related by Dr. Greene, but every body knows that it is present, also, in almost all cases of disease of the heart, when at all advanced in duration or extent. We recollect an instance of hypertrophy and dilatation of the ventricles, attended with slight dilatation of the ascending arch of the aorta, in which the dyspnœa was more distressing than we ever, before or since, observed it. Yet, in that case, there was no mechanical pressure exerted on the lungs or on the bronchi.

There can be no question that an accurate acquaintance with the general symptoms of affections of the heart and arteries, is indispensable. The acute observer, if an accurate pathologist, will often at a glance, at the mention of a symptom, or a few symptoms, have a train of suspicions and reflections excited, which will lead him, by a rapid process, to a close approximation to the truth. How frequently does a patient present himself, with the idea that he is labouring under indigestion. He complains excessively of flatulence and oppression at the præcordia; but the puffy face, the short breath, and the strong pulse, point at once to the heart or the larger bloodvessels as the probable seat of the affection. The physiognomy and the general features of disease are subjects of high interest, and the utmost consequence to the practical surgeon or physician. It is the power of deciphering these characters with rapidity which confers that diagnostic acumen, that more, perhaps than any other quality, leads the possessor to reputation and success.

Dr. Greene observes, with reference to dysphagia, as a symptom of thoracic aneurism, that:—

"The difficulty of swallowing is more likely to be experienced in those cases where the disease occupies the transverse and descending portion of the arch, than elsewhere. In this situation the œsophagus is more fixed, as it were, than in any other portion of its course, except where it passes through the diaphragm. From its comparative mobility, in the upper and lower portion of its course, it may in a great measure elude, for a considerable time, the pressure of the tumour. When this symptom does occur, it only becomes valuable when taken in conjunction with the physical signs, and of course cannot be relied upon if it occurs as an isolated phenomenon. The importance, in such a case, of being able to ascertain its dependance on the pressure of an aneurismal tumour, is obvious from the fact of probangs having been passed down in this disease, under the supposition that the stricture was unconnected with it."

He remarks the turgescence of the left jugular vein, which occurred in each of the three cases recorded. This general symptom is so fallacious, that we would not place much dependence on it. Turgescence and engorgement of one or both jugulars is so frequent in various affections of the heart, and so little of certainty seems to attend its explanation, that, although a suspicious general feature, it is no satisfactory index of the actual nature of the alteration.

Dr. Greene very properly dwells on the necessity for studying all symptoms and all signs in connexion with each other. Diagnosis, in obscure or doubtful cases, is a calculation of probabilities—the more circumstances are omitted, the more likely the calculation is to be erroneous. Hence we see the blunders very frequently committed by men of eminence in the profession. Trusting to their experience, they seize upon one, or a very few symptoms, and, snatching an opinion on these insufficient data, they commit some palpable mistake.

The result of every-day observation and experience, and the study of recorded cases, combine to prove, that aneurism, seated in the arch of the aorta, is distinguished with infinitely more facility than when the tumor is seated lower down. The reasons are so obvious, that we need not dwell upon them. Aneurisms of the lower part of the thoracic aorta are frequently, perhaps they are generally overlooked. As yet, we have no satisfactory symptoms nor physical signs to determine their existence. Let us hope that accurate, very accurate observation will do something for us.

Dr. Greene offers what we must denominate suggestions for a diagnosis. They are these :—

" 1st. An impulse limited in extent, and decreasing in intensity as the stethoscope is applied above or below the situation of the suspected tumor. This sign is more valuable, if it occurs on the right side of the spinal column, which is not the situation, anatomically speaking, where an impulse should be perceived.

2d. A bruit de soufflet or a bruit de râpe, heard in the suspected tumor, and not observable in any other considerable portion of the aorta or in the heart. It is necessary in every instance to examine the heart, for within these few days, I have met with a case, in the Whitworth Hospital, where a loud bruit de râpe accompanies the second sound of the heart, this bruit is heard down the spine along the course of the aorta. It is heard also in the carotid and brachial arteries, even to the elbow-joint. I have satisfied myself that in this case a double sound is heard in these arteries. The name of the individual in whom these phenomena occur is William Connor, and several of the class of the Whitworth Hospital have heard both the bruit and the sounds in the situations mentioned.

3rd. The production of pain on pressing the vertebral column over the site of the impulse.

4th. The production of pain referred to a portion of the lung near the situation of the impulse, on a forcible inspiration.

5th. Dysphagia referred to some point below the middle third of the sternum, or to a point nearly opposite the impulse.

Lastly, it may be worth while to contrast the pulsations of the abdominal aorta in the epigastrium, with other arteries and with the impulse of the heart.

We should take into consideration also (provided the above signs are well marked) the evidence we may obtain with respect to the position of the tumour from the absence of some symptoms; such, for instance, as the non-existence of a laryngeal croupy cough; difference of pulsation in the two radial arteries, or numbness or œdema of the upper extremities."

We think we have said enough upon this subject. We would intreat the attention of practitioners to it. Diseases of the heart and of the larger arteries are extremely frequent; but, what is singular, they are as frequently mistaken. Scarcely a week passes, but we see some patient in whom cardiac disease, or enlargement of the arch of the aorta, has been overlooked —a remarkable and almost unpardonable error, considering the advances that have lately been made in the physical means of diagnosis.

---

### Traumatic Tetanus Cured.

There are so few instances of recovery from this dreadful disease, that it is consolatory to be able to relate a fortunate termination. This case is related in the 5th vol. of the Calcutta Transactions, by Mr. Grant. The patient was a Parsee, who had received a slight contused wound in the face, and a fortnight afterwards exhibited unequivocal symptoms of trismus. Some mercurial ointment and camphor had been rubbed on the side of the face before entrance into hospital. The pulse was quick, but there was no other constitutional disturbance. One hundred drops of laudanum were given him at bed time—the warm bath—belladonna plaster. Next day he seemed a little better, and a quill could be got into the mouth to inject nourishment. Fifty drops were given the second night, the bath to be repeated. A turpentine injection was ordered to obviate the constipating effects of the laudanum. Next day he could open the jaw half an inch. Medicines as before. On the fourth day he retrograded much. He was ordered three grains of extract of belladonna. Fifth day, the pupils dilated. The medicine to be repeated, and to have an enema twice, each containing fifteen grains of tartrite of antimony. *Sixth day.* The injections had produced a general feeling of coldness and weakness, but no vomiting. The second enema was followed by two motions. Could not swallow pills. Sixty drops of laudanum to be given. To have the warm bath *usque ad deliquium.* These remedies, with some variation, were continued several days. On the 12th day opisthotonos occurred, and rendered the case more hopeless. Was ordered two grains of extract of stramonium every two hours. This seemed to afford relief, and the medicine was continued in smaller doses. On the 18th day, the stramonium was omitted, and ten grains of calomel with three grains of opium were given. The bowels were kept open by enemas. He continued to mend, though slowly, from this date.

Another case somewhat similar, is related in the same volume, by Dr. Gilmore, where tetanus followed a severe sword-cut in the thigh. This patient was freely bled after the spasms had commenced—and the treatment consisted chiefly of calomel, camphor, soda, and purgatives. One of these purgatives produced vomiting and purging, during which large indurated masses of fæculent matter were discharged. From this time the symptoms became more favourable, and recovery took place.

### Tetanus Treated by Tartarized Antimony.

This case is related in the Dublin Journal for July last, by Mr. Woodward. The patient was a labourer, and was found by Mr. W. labouring under all the symptoms of tetanus, opisthotonos. These had been gradually coming on for two days. No other cause but exposure to cold and wet could be assigned. He had been bled by an apothecary, without relief. Mr. W. first ordered him two grains of calomel with eight of Dover's powder, every three hours—blister—turpentine enema, &c. The symptoms became worse. An antimonial solution (a grain to the ounce) was given in doses of a dessert spoonful every hour, unless considerable sickness ensued. A turpentine injection brought away much black and fetid fæces. The pulse fell—much weakness was produced—the muscular rigidity diminished—and he swallowed better. The doses of the antimony were lessened—and the patient gradually recovered.

---

### On Typhoid Pneumonia. By Dr. Hudson.

Inflammation of the lungs, accompanied by a typhoid fever, instead of a strong inflammatory one, is a disease well known to practitioners in the army and navy, who had seen much service last war. In the Winter time, and after disasters by flood and field, pneumonia typhodes was a very common and a very fatal disease. The French prisoners, in the different depôts, suffered severely from it, and so did our troops after the retreat of Sir John Moore from Spain. In addition to the usual symptoms of pulmonic inflammation, and those of an aggravated character, we have, in this disease, great prostration of strength, feeble, rapid pulse, head-ache, delirium, ardent thirst, and sometimes bilious vomiting, partial cold sweats, diarrhœa, black, dry tongue, fetid breath in bad cases, with thin and sanious, or sanguineous expectoration. Its treatment is very difficult, as it will not bear well that depletion which is necessary to quell the local inflammation; and yet, if the pulmonic inflammation is not subdued, the consequences are fatal.

"The *physical signs* are occasionally perfectly latent, frequently partially so; and from a careful observation of a very considerable number of cases, this latency has seemed to me to bear a certain relation to the symptoms, or rather the pathology, which would lead to a threefold division into—

1. Cases in which the typhoid type was connected with a congestion of the lung, independent of inflammation; the effect of which was to cause the signs of the latter to become latent either wholly or in part.

2nd. Cases in which this latency co-existed with gastritis, the typhoid type depending probably on the latter.

3rd. Cases of typhoid pneumonia, with more intense gastritis, and without modification of signs.

I am indebted to the kindness of Dr. Stokes, for permission to make use of the reports which I furnished of a number of cases which occurred in the Meath Hospital, in the early part of 1833, when I acted as his clinical clerk. The remainder of the cases fell under my own care in the Navan Fever Hospital, during a recent epidemic of typhoid pneumonia."

Several cases in illustration are minutely detailed, and deserve attentive perusal, in the Dublin Journal, where they are published. We give the following extract respecting the *latency of physical signs.*

"I shall offer a few remarks on the *latency of signs* sometimes met with in the *foregoing* and other cases of this disease. This consisted principally in the absence of what is considered by some the pathognomonic sign of the first stage, crepitating râle; sometimes even in that of the second also, bronchial respiration. It has been observed, that in some instances where a few hours before no signs of pneumonia were present, dulness on percussion and bronchial respiration were found established, in others a degree of dulness on percussion, and feebleness of respiration, denoted the existence of a congestion which slid into solidification, without the signs of the first stage intervening. My inference from these facts is, 'that the view of those who suppose that this disease is essentially a complication of pneumonia, with a superadded passive congestion,' is correct; and I would answer the objections which may be made to the truth of the fact, and the validity of the inference, in this way. It, 'the crepitus,' had not *passed* before I saw the patient, as the disease sometimes seemed almost to begin, and commonly progressed under my observation, and that of others. My examinations were purposely so frequent, that it could scarcely have escaped in the mean time, and yet the disease was traced onwards from a very small portion, till nearly the entire organ was engaged in some cases without crepitus being once heard, and in others a mere trace of it, until resolution or softening commenced. Then as to the inference; the proof that the disease is a passive congestion rests not merely on the signs, but also on the general symptoms and appearances on dissection, and is strengthened by the observation of the effects of bloodletting; these were strongly marked in Malone (March 3rd), but even more so in the following case, a brief report of which I shall abstract from my note-book."

In two of the cases detailed by our author, there was a remarkable phenomenon—"*tympanitic clearness, on percussion, over a solidified lung, without air being present in the pleura.*" This clearness of sound cannot be described in words. It is sometimes as clear as in cases of pneumo-thorax. It cannot be confounded with the sound of the healthy side of the chest—and it is still more different from that of a hepatized lung. The following were the auscultic signs when Case 7 was brought into hospital. The pulse, meantime, was 132, and the respiration 60 in the minute.

"Signs: on percussion the right side anteriorly gave a healthy sound; the left *a sound of precisely the same kind as that produced by percussing over the stomach or cæcum;* posteriorly there was dulness over the spine and upper part of the scapula; respiration was tracheal under the clavicle; null below and over most of the anterior of this side; a very feeble crepitus was heard, with strong resonance of voice; over the entire side, posteriorly, a very feeble murmur of respiration, nearly quite inaudible in the upper portion; bronchitic râles over the right side."

Dr. Hudson having met with four cases of this kind, where dissection was permitted, he gives a short account of the appearances POST MORTEM.

"The first, which at the time surprised me not a little, was that of a man named Drillon, who died of extensive inflammation of the left lung in the Meath Hospital in the spring of 1832.

At the close of this case, from the *hollow* sound on percussion of the lower part of the left side (previously quite dull), a pretty general opinion existed, that a pneumonic abscess had formed, and burst into the pleura. The side was punctured accordingly, but no air escaped, and farther dissection shewed the pleura adherent to two-thirds of the lung, red and solid, but *no abscess*. The next was a man who presented himself at the same hospital, with the history and symptoms of phthisis, and on percussion under the right clavicle, there was such a remarkable muffled tympanitic sound, with tracheal respiration and resonance of voice, that all who heard these phenomena expected to find a large tuberculous cavity; dissection however, shewed the lung hard and solid throughout from *chronic pneumonia*. The other two cases of Malone and Smith were similar to that of Drillon. It seems to me very obvious, that these cases cannot be brought under the explanation given by the high authorities just quoted, and I confess myself quite unable to give one which would be considered more satisfactory."

We think this phenomenon is one of the most puzzling which we have ever heard of in our difficult art!

In respect to treatment, Dr. H. saw the bad effects of free bleeding. Local bleeding was beneficial—but the most successful practice was by means of calomel in large doses. "The least I can say of this mode of giving mercury in inflammation is, that it has never disappointed me, though it has often surprised me." The dose was a scruple, repeated in six hours. The following is the chief and last inference which Dr. H. draws.

"4th. That the epidemic constitution of the disease, and its pathological conditions (connected as both seem to be, with an altered state of the blood), often combine to render these cases prone to run into gangrene, and that they are otherwise tedious and intractable, and generally require the early and liberal use of stimulants, as well as the prompt but cautious employment of the most active means for subduing inflammation; and, I think, the necessity for the former may be stated to be in proportion to the degree of prostration: and the extent of passive congestion as indicated by dulness on percussion and nullity of all respiratory phenomena."

The paper is very creditable to Dr. Hudson, and has recalled our minds to many a harassing and distressing scene which we have witnessed in the late war, both at home and abroad!

---

A Series of Twenty Plates, illustrating the Causes of Displacement in the various Fractures of the Bones of the Extremities. By G. W. Hind, M.R.C.S. Formerly House-Surgeon to the Middlesex Hospital, late Curator to the Museum of Anatomy in the University of London. Lithographs, folio, bds. London, 1835.

The object of this very meritorious series of Lithographic Plates is, as our readers may observe from the title-page, to exhibit, in a plain and striking manner, the causes of displacement in the various fractures of the bones of the extremities. The work is appropriately dedicated to Sir Charles Bell, whose pupil the author formerly was.

In the first plate are shewn the causes of the displacement of the clavicle, in fracture of its shaft; the subclavius muscle is accurately exhibited, drawing the outer portion downwards and inwards.

In the second plate, the coracoid process is seen broken, and its extremity drawn downwards and inwards, by the short head of the biceps, the coraco-brachialis, and the pectoralis minor.

In the third plate, the fractured extremity of the acromion is drawn downwards and forwards by the deltoid muscle.

In the fourth plate, the neck of the scapula is broken. The glenoid portion is carried downwards by the weight of the arm, yet the glenoid cavity still remains applied to the head of the humerus, by the action of the long heads of the biceps and the triceps.

In the fifth plate, the neck of the humerus is fractured. The principal displacement results from the pectoralis major, the latissimus dorsi, and the teres major drawing the upper extremity of the shaft of the bone inwards.

In the sixth plate, we observe fracture of the humerus between the insertions of the axillary muscles and that of the deltoid. The former muscles pull the upper portion inwards, while the deltoid drags the lower portion outwards.

In the seventh plate, the fracture is below the insertion of the deltoid. The displacement here depends greatly on the direction of the fracture.

In the eighth plate, the fracture of the humerus has occurred immediately above the condyles. The displacement is considerable, and simulates dislocation backwards of the radius and the ulna. The lower portion glides backwards over the broken surface of the upper portion by the combined actions of the brachialis anticus and biceps, which are situated on the anterior part, and by the triceps which is on the posterior part.

In the ninth plate, there is fracture of the coronoid process of the ulna. This process is drawn up by the brachialis anticus. The olecranon process is also seen fractured, and drawn upwards by the triceps.

In the tenth plate, the neck of the radius is broken above the insertion of the biceps. This pulls the lower portion forwards, upwards, and inwards. The radius is also drawn broken near the centre.

The eleventh plate portrays fracture of the shaft of the ulna, and fracture of the radius and ulna near the centre. The pronator quadratus, in both, plays the most important part, in causing the broken bone or bones to approximate.

In the twelfth plate, we are shewn fracture of the lower ends of the radius and ulna. This is a fracture often mistaken, and difficult to treat; yet it is far from being uncommon. The appearances greatly resemble those of dislocation of the carpus backwards. The force being generally applied on the palmar aspect, the lower portions are drawn upwards and backwards by the combined action of the flexors and extensors. Perhaps, however, it is the superior power of the flexors which tends to this effect.

In the thirteenth plate, we descend to the lower limbs. The superior portion of the trochanter major is broken off, and drawn up by the glutæus medius and minimus.

In the fourteenth, we have fracture of the neck of the thigh-bone within the capsule. The mass of rotators, the glutæi, and the muscles of the thigh, combine to draw up and evert the shaft of the femur, and the limb.

In the fifteenth plate, there is drawn fracture of the femur, below the trochanter minor. The upper portion, comprising the trochanter major, is drawn upwards, nearly at a right angle by the action of the muscles inserted into the trochanter minor—the psoas and iliacus.

The sixteenth plate displays fracture of the shaft of the femur, near the centre. The lower portion is drawn upwards and backwards, chiefly by the operation of the adductors.

In the seventeenth plate, there is fracture of the femur, immediately above the condyles. The lower portion is drawn backwards by the gastrocnemius, plantaris, and popliteus.

In the eighteenth plate, are delineated fractures of the patella.

In the nineteenth, we find fractures of the tibia and fibula. The displacement depends greatly on the direction of the fracture.

In the twentieth, the last plate, we are presented with fracture of the inner malleolus, and Pott's fracture of the fibula.

We have run over the subjects of these several plates. It remains for us only to speak of their execution. This is bold, pictorial, and correct. It is seldom that we see such vigour in anatomical plates. Mr. Hind is a first-rate draughtsman, and has caught his inspiration from the masterly pencil of his preceptor, Sir Charles Bell. Altogether we think the work both admirably executed and extremely useful. To our young friends it will be a very valuable acquisition—to many of our old ones it would be highly useful. We have placed it in our own library.

